# MicroEye : A low-cost online tool wear monitoring system with modular 3D-printed components for micro-milling application

**DOI:** 10.1016/j.ohx.2022.e00269

**Published:** 2022-02-15

**Authors:** Christiand, Gandjar Kiswanto, Ario Sunar Baskoro, Fachryal Hiltansyah, Muhammad Ramadhani Fitriawan, Ramandika Garindra Putra, Shabrina Kartika Putri, Tae Jo Ko

**Affiliations:** a Department of Mechanical Engineering Universitas Indonesia, Jawa Barat, Indonesia; b School of Mechanical Engineering Yeungnam University, Kyoungbuk, South Korea

**Keywords:** Micro-milling, Tool wear monitoring, Vision, 3D-printed component, Low-cost

## Abstract

Tool detachment during the machining process is often required by many image-based tool wear monitoring (TWM) systems. Tool detachment prevents the online mode of the wear measurement, extends the machining time, and contributes to measurement inaccuracy. Other alternatives of the image-based TWM systems have been developed with the image-acquisition device located statically near the tool position without the requirement for the tool detachment. However, due to its proximity to the machining site, the image-acquisition device may experience obstruction from the workpiece chips and the splash of coolant fluid during the machining process, resulting in non-optimal TWM. This article presents MicroEye – an online image-based TWM system with modular 3D-printed components to overcome the two problems. MicroEye offers great flexibility in its operation through the use of an active 6-DOF (degree of freedom) robotics arm with a camera at the end-effector. MicroEye does not require tool detachment to perform tool wear monitoring and can be safely placed outside the machining area. MicroEye is the first open-sourced, 3D-printed components and active dynamic-type TWM system for the application of micro-milling. MicroEye can be built at a low-cost (approximately US$ 872, including the camera). MicroEye is suitable for various micro-milling sites, from laboratory scale to middle-low workshop.

## Graphical abstract

**Figure:**
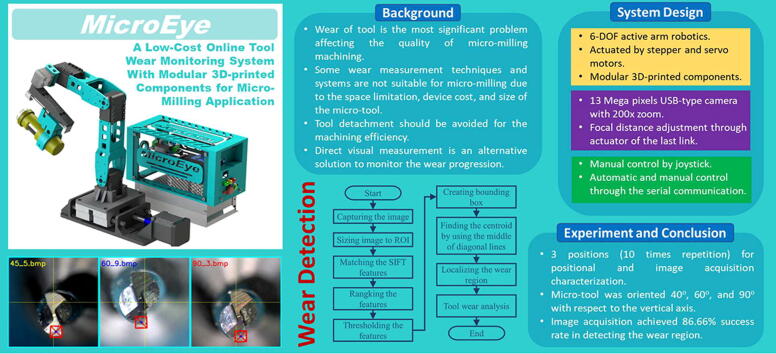

## 1 Hardware in context

Tool condition monitoring (TCM) has been researched for more than two decades, primarily for general machining processes such as macro-milling. The growing demand for micro-products has attracted researchers to put more effort into the TCM developments for the micro-milling domain. Tool wear monitoring (TWM) is a subset of the TCM field, which takes the wear of the tool as the object of the monitoring process. The tool wear itself is a physical condition occurring on the tool surface, particularly in the cutting edge area. Several image-based tool condition monitoring (TCM) systems for the macro and micro-milling have been developed in the past [1], [2], [3], [4], [5], [6], [7]. Such TCM systems with a direct measurement approach can capture the wear occurrence better than the indirect measurement (such as by using force, vibration, or acoustic emission sensors) since the image series of the tool surface can show directly whether the wear has progressed or not.

**Table:** 

**Specifications table:**	
**Hardware name**	MicroEye
**Subject area**	Manufacturing, Engineering, and Material Science
**Hardware type**	- Tool wear monitoring system
	- Mechanical engineering and materials science
**Open source license**	CC-BY-SA-4.0, GNU GPL
**Cost of hardware**	US$ 872
**Source file repository**	https://doi.org/10.17632/w26x9yrbtm.2

Szydłowski et al. have developed a machine vision consisting of two CCD cameras and a motorized spindle to get the surface images of the micro-milling tool from various viewing angles [1]. The algorithm of their machine vision used depth information with variable light intensity to reconstruct the wear image. The micro tool was detached from the machining center and placed to the motorized spindle during the image acquisition. Dai et al. constructed a machine vision for micro-tool wear inspection with a 3D-positioning module [2]. The dedicated positioning module guaranteed that the micro-tool images were taken without tool detachment to preserve measurement accuracy and validity. However, the image-acquisition device with its positioning module occupied a significant portion of the machining center working space. Fernandez et al. has proposed the image processing procedures to detect the worn area for micro-tool in micro-milling [3]. The procedure was based on morphological operations, *k*-means clustering, and Otsu Multilevel algorithm. The result showed 5% differences between the predicted and actual worn area. However, as far as the online measurement mode was concerned, the author did not explain the image-acquisition system or procedures with the details, nor the information about the tool detachment. Malhotra et al. presented the algorithm to detect the tool wear in micro-milling based on color segmentation with Fuzzy clustering [4]. The image acquisition was performed by using a CCD camera microscope with HD full resolution. In every 300 mm machining distance, the micro-tool was cleaned in an ultrasonic bath for 15 minutes using acetone medium. Thus, the tool detachment was needed. The system achieved overall accuracy 97% in detecting the flank wear. In the field of macro-milling, Zhang et al. has developed a machine vision system based on the illuminated CCD camera with light-emitting diode (LED) ring attached [5]. The whole image-acquisition module was placed on the workpiece table beside the workpiece jig. The image of the tool flank was acquired by actuating the workpiece table so that the tool flank was contained in the viewing region of the CCD camera. The effort to enhance the robustness of machine vision in measuring the tool condition in macro-milling has been made by Fernandez et al. [6]. The machine vision was developed to detect the broken insert in the profile milling application. Their work was focused on building the data set and enhancing the cutting edge localization algorithm. They claimed that their system was capable of the online mode of wear measurement without tool detachment (at the tool resting position). However, the structure of the image-acquisition system for the online measurement was not explained and left for their future works. That would be a crucial issue since the online measurement should consider the feasibility of the image-acquisition system placement at the real machining site and the avoidance of obstructing materials such as coolant fluid and workpiece chips.

For the optimal online TCM in real machining environments, the system should ideally be dynamic and not require tool detachment. Firstly, dynamic means that the TCM system can be actively operated anytime with arbitrary motion path and position. With such ability, the TCM system can be integrated into the existing machining plan without alteration to the existing tool path. Furthermore, the image-acquisition position can be executed on-demand anytime so that the system does not collide with the other objects in the machining area. Secondly, the tool detachment should be avoided for machining efficiency and measurement accuracy. Indeed, the tool detachment affects the machining efficiency by extending the machining time and adding more procedural steps. From the measurement point of view (especially for the experimental works), tool detachment can microscopically add random error to the measurement since the installation condition before and after the tool detachment might not be the same. Moreover, the measurement quality will degrade if no appropriate standard of procedure in tool attach/detachment is followed. To the author’s knowledge up to date, very few image-based TCM systems address the issue of tool detachment and system structure (dynamic or static). That can also be concluded by examining all the TCM systems developed prior to the year 2014 in the list of “Table 1. DirectTCM techniques based on image processing.” in the review report by Dutta et al. [8]. The TCM systems can be categorized into: (**a**) the static system without tool detachment – 10 systems, (**b**) the static system with tool detachment – 15 systems, (**c**) the dynamic (actively movable) system with tool detachment – 2 systems. None of the listed systems is dynamic-type without tool detachment feature. Therefore, research is needed to develop an image-based TCM system with dynamic and non-tool detachment features.

**Fig. 1:**
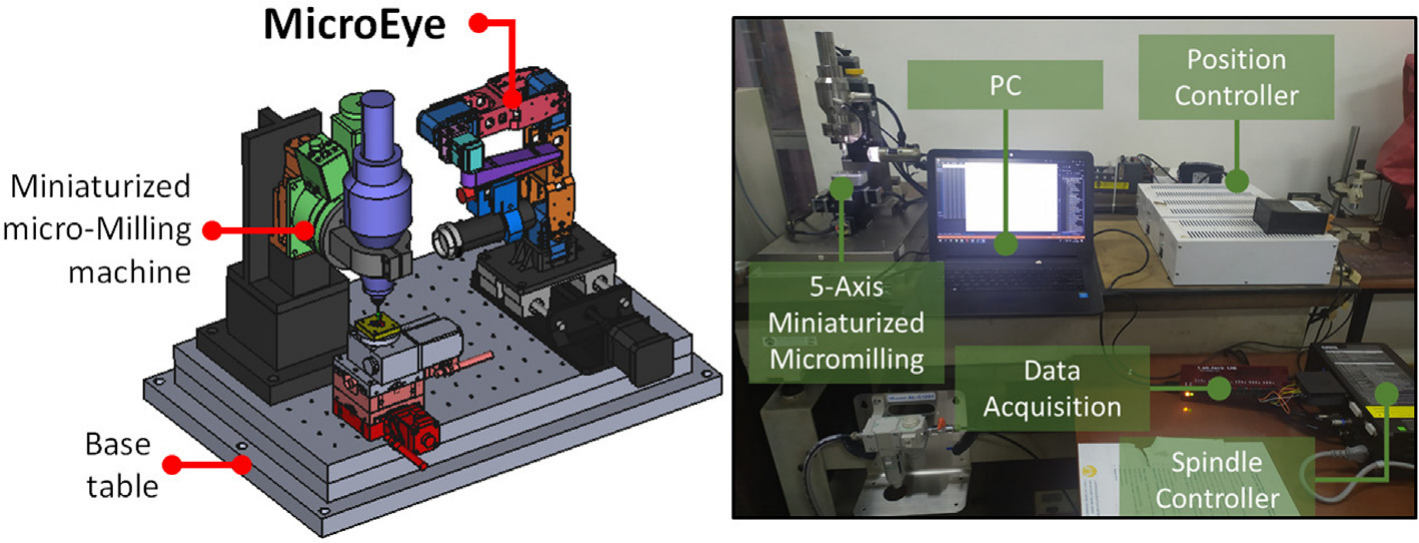
Overview of MicroEye installed at miniaturized micro-milling environment.

In this paper, a TWM system for micro-milling called MicroEye is presented (Fig. 1). MicroEye was developed to provide an alternative solution for the tool wear monitoring in the micro-milling application. MicroEye was designed as an online TWM system with the active 6-DOF robotics arm and the image-based wear detection technique. The paradigm of MicroEye development is the rapid creation of a low-cost open-source TWM system by utilizing 3D digital printing technology. The system consists of two major parts, i.e., positioning mechanism and vision sensor. The tool wear during micro-milling operation is assessed using computer vision to detect and analyze the wear region from the tool images.

## 2 Hardware description

MicroEye is a TWM system with a direct visual measurement approach. MicroEye directly measures the tool wear based on the images of the cutting tool surface. MicroEye consists of a 6-DOF (degree-of-freedom) positioning system serially constructed from six 3D-printed links. The microscope camera as the vision sensor is attached to the positioning system’s end-effector (last link). 6-DOF positioning system gives freedom in locating the camera to any positions that produce the best viewing angle for image acquisition. Another point of view, the 6-DOF positioning system also gives more freedom for the users in selecting the location of MicroEye base at the limited area of micro-milling environment, while at the same time keeping the same desired viewing angle of the camera. During the monitoring process, the cutting tool is not necessarily detached from the collet when the camera captures the images of the tool surface. Tiny position changes of the tool are avoided due to the absence of tool detachment, which guarantees that every measurement point in the one run of the micro-milling process is observed continuously with the same environment and machining condition.

MicroEye brings the concept of modular, 3D-printed, low-cost, and vision-based TWM. The entire system of MicroEye is assembled from the nine modules available. The modules are base, first link to sixth link, microscope camera, and controller unit. The connection between modules is designed carefully for the ease of the user. The structure and body of links are 3D-printed to foster customization, open-source, and rapid fabrication. The 3D-printed components of MicroEye are lower in fabrication cost compared to metal-based components. The microscope camera is a low-cost USB microscope type widely available in the market with 200 times magnification factor.

### 2.1 Positioning system

**Fig. 2:**
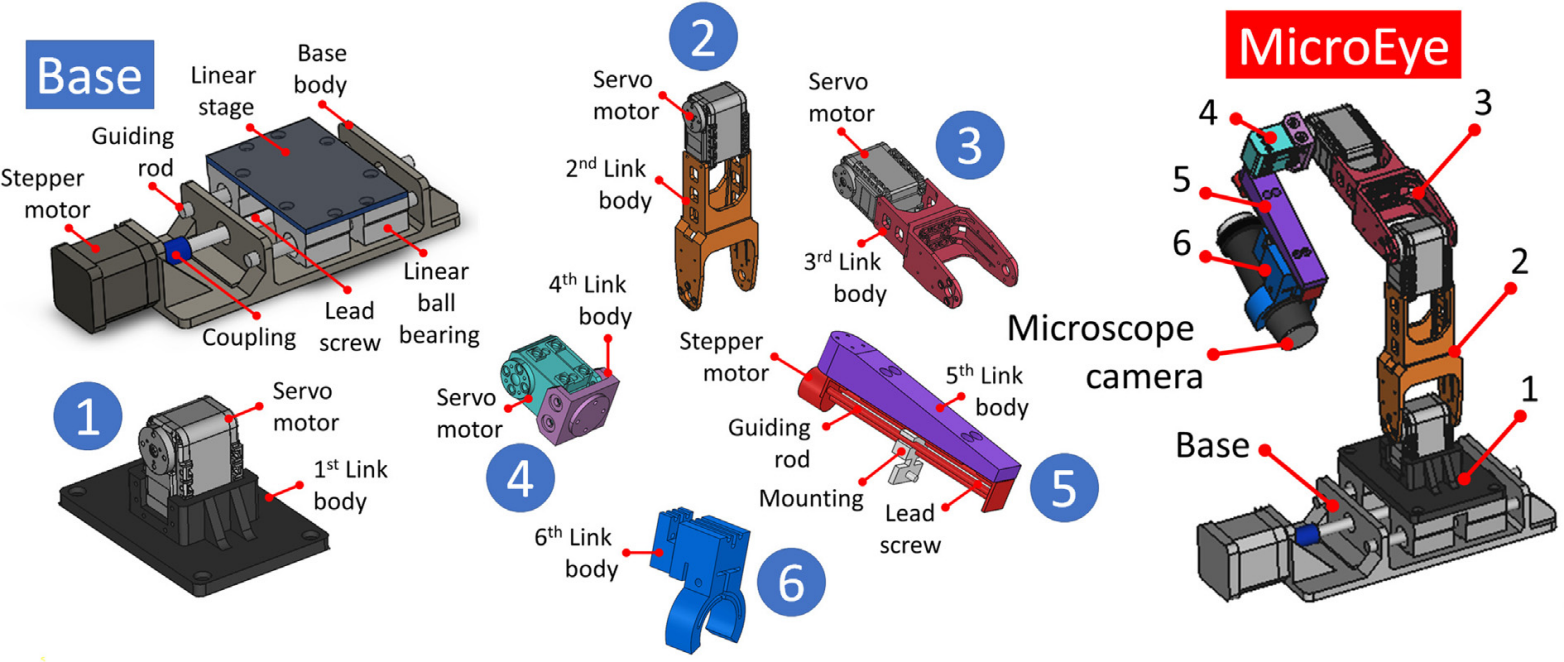
The MicroEye design.

The positioning system holds the microscope camera and achieves the desired viewing angle for image acquisition. The positioning system is movable since a DC motor actuates every joint. The stepper motor is for the first and last joints. The servo motor is for the second joint until the fifth joint. The kinematics of the positioning system follows PRRRRP (Prismatic-Revolute-Revolute-Revolute-Revolute-Prismatic) joints arrangement. The structure of the whole positioning system can be seen in Fig. 2. The exploded view of the design shows the components from the seven modules of the positioning system.

#### 2.1.1 Base

The base is the main support for the whole system of MicroEye. The base body is a 3D-printed component with PLA (PolyLactic Acid) material. The base contains a NEMA-17 stepper motor as the actuator of the first joint. In order to produce linear motion, the motor shaft is connected to the lead screw through a flexible coupling. The lead screw has a 1.5 mm pitch and 150 mm total length. Two guiding rods provide motion support for the linear stage of the MicroEye first link. The effective range of linear motion is 30 mm.

#### 2.1.2 First link

The first link body is PLA 3D-printed component. The body is attached to the commercially available metal linear stage. The servo motor as the second joint actuator of MicroEye is mounted to the body. The servo motor type is AX-12A made by Dynamixel and can rotate 300 °.

#### 2.1.3 Second and third links

The body of the second and third links have the shape of a lever bar from PLA 3D-printed component. The servo motor is attached to one end of the body for the actuation of the next joint respectively. The motion of the second and third links are rotational, driven by the motors from the respective previous links. The servo motor type is AX-12A made by Dynamixel and can rotate 300 °. The mounting design for placing the servo motors is modified from CAD files provided by the manufacturer [9].

#### 2.1.4 Fourth link

The fourth link merely provides mounting for the servo motor XL-320 made by Dynamixel. The fourth link, together with its joint, is intended to give more capability to the system in orienting (α,β,γ) the viewing angle of the microscope camera. In contrast, the first to the third joint achieves the desired location (X, Y, Z) of the observation point. XL-320 is faster and has a more compact size than AX-12A. The XL-320 is suitable for higher joints due to its size, weight, and lower power consumption. The body of the fourth link is PLA 3D-printed.

#### 2.1.5 Fifth link

A lead screw actuated by a micro-stepper motor is attached to the body of the fifth link. The lead screw provides the capability to adjust the focal distance of the microscope camera. The forward and backward movement of the lead screw gives control for the user in adjusting the focal distance of the microscope camera. The motor can move 0.25 mm in one step with a maximum linear speed of 2.5 mm/s. The lead screw movement is the actuation of the sixth joint. The body of the fourth link is PLA 3D-printed.

#### 2.1.6 Sixth link

The sixth link has the function of holding the microscope camera. The link is attached to the movable mounting at the lead screw of the sixth joint. The gripping power can be adjusted by turning the two bolts on the left and right sides of the link.

### 2.2 Microscope camera

The microscope camera is an AD4113ZT USB-type camera with 200x magnification and resolution maximum at 1.3 megapixels (1280 × 1024) by Dino-Lite. The microscope camera is considered a decent and low-budget vision sensor compared to other solutions for digitally capturing tool surface conditions, such as scanning electron microscope (SEM) or desktop digital microscope. From the author’s experience, the USB-type microscope camera is adequate to capture the wear areas of the micro-tool. However, the wear detection from the tool images should be accompanied by a good lighting environment and computer vision techniques.

### 2.3 Controller unit

**Fig. 3:**
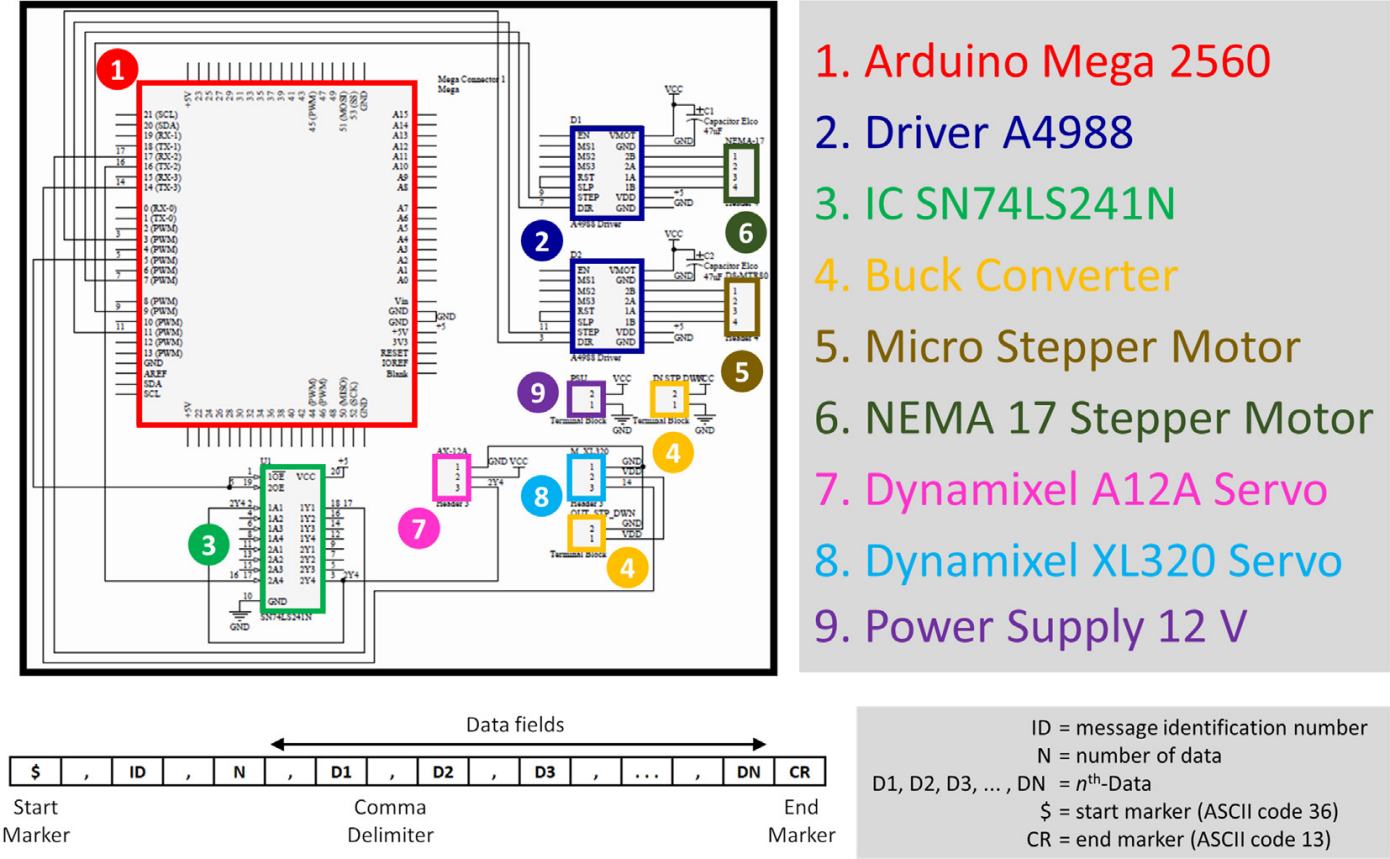
The wiring schematic of the controller unit and the command through serial protocol.

The controller unit of MicroEye consists of the main controller and the six motor drivers. The role of the main controller is to receive commands from the externals, e.g., computer running the program of the micro-milling process sequence, joystick, or any serial terminal program for manual operation. Arduino Mega 2560 was chosen as the main controller for MicroEye. Six motor drivers are connected to each motor, depending on the motor type. The stepper motors of the first and sixth joints are driven using the A4998 driver module with a sixteenth-step division at maximum. The AX-12A servo motors of the second, third, and fourth joints are driven by using IC 74LS21N with an additional wiring scheme. The Arduino Mega 2560 and all motor drivers are integrated into one controller printed circuit board (PCB) for compactness. The schematic of the board wiring is presented in Fig. 3. The controller PCB is contained in the box enclosure to ensure the controller unit’s safety and durability from external disturbances.

The communication between the main controller and externals is facilitated by using serial communication. The externals send the ASCII string command to the main controller in the specified format as shown in Fig. 3. For example, if the user wants to move the MicroEye to configuration: 1^st^ joint  = 5 mm, 2^nd^ joint  = 20°, 3^rd^ joint  = 30°, 4^th^ joint  = 40°, 5^th^ joint  = 50°, 6^th^ joint  = 6 mm, the command would be ”$,2,6,5,20,30,40,50,6*\*r” (without quote marking).

### 2.4 Kinematics

#### 2.4.1 Forward kinematics

**Fig. 4:**
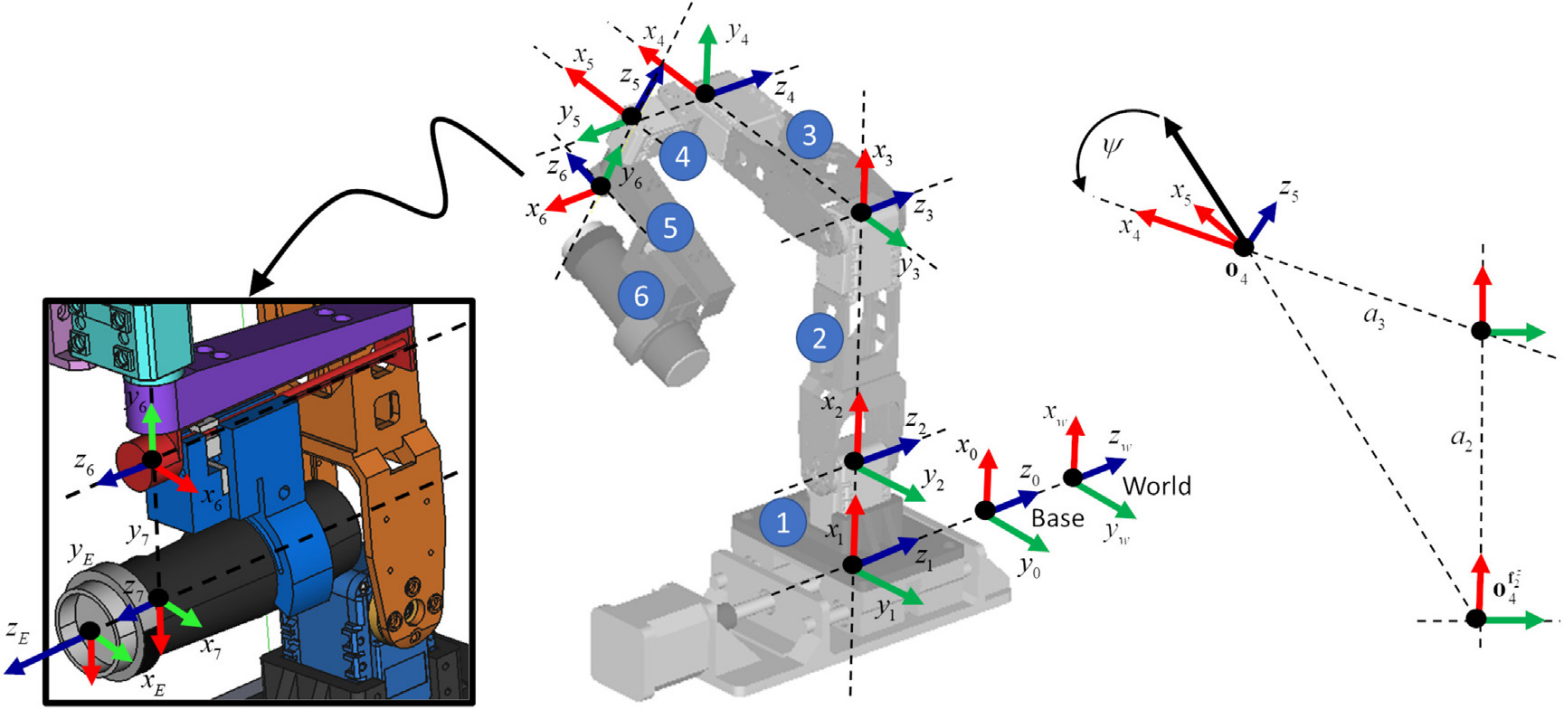
Joint coordinates assignment for kinematics derivation.

In order to define the forward kinematics of MicroEye, six joint coordinates are assigned to the joints of MicroEye by following the DH (Denavit-Hartenberg) convention [10] (Fig. 4). The *i*-th joint coordinate ($F_{i}$) is represented by a 4x4 homogenous transformation matrix. The joint coordinate also acts as the reference frame coordinate for the (*i-1*)-th link with respect to the world coordinate ($F_{\mathrm{world}}$). The DH parameters parameterize the relative position between two consecutive joint coordinates. The joint coordinate with respect to the world coordinate is formally defined in Eq. (1) with the DH parameters for each joint listed in the Table 1.

(1) $$F_{i+1}=F_{i}\left[\begin{array}{cccc} \cos\theta_{i} & -\sin\theta_{i}\cos\alpha_{i} & \sin\theta_{i}\sin\alpha_{i} & a_{i}\cos\theta_{i}\\ \sin\theta_{i} & \cos\theta_{i}\cos\alpha_{i} & -\cos\theta_{i}\sin\alpha_{i} & a_{i}\sin\theta_{i}\\ 0 & \sin\alpha_{i} & \cos\alpha_{i} & d_{i}\\ 0 & 0 & 0 & 1 \end{array}\right]\text{;}\;i\in \left[1,2,3,4,5,6\right]$$

For the base link, the reference frame coordinate is denoted as $F_{\mathrm{base}}$ (a.k.a. $F_{0}$). The reference frame coordinate and the world coordinate can be chosen arbitrarily depending on the machining environment. In this work, the $F_{1},F_{\mathrm{base}}$ and $F_{\mathrm{world}}$ are made coincidence to the $F_{2}$ for the convenience of kinematics calculation. Therefore, the relationship between $F_{\mathrm{world}},F_{\mathrm{base}}$, and $F_{2}$ is shown in Eq. (2).

(2) $$F_{\mathrm{world}}=F_{\mathrm{base}}=F_{1}=F_{2}=I^{4\times 4}$$

A coordinate reference frame for the end-effector ($F_{E}$) should be located at the most intuitive location for the user to set the image-acquisition setting, i.e., at the most front of the camera lens (Fig. 4). The coordinate reference frame for the end-effector can be calculated by using Eq. 3. However, it should be noted that the transformation matrix from $F_{7}$ to $F_{E}$ can be different if the type of the camera, the lens radius, the mounting location are different from the one used in this work. Thus, the transformation matrix should be adjusted for different cases, particularly to update the $d_{f}$ distance between the end-effector origin point and the $F_{7}$ origin point.

(3) $$F_{E}=F_{7}\left[\begin{array}{cccc} 1 & 0 & 0 & 0\\ 0 & 1 & 0 & 0\\ 0 & 0 & 1 & d_{f}\\ 0 & 0 & 0 & 1 \end{array}\right]$$

**Table 1:** DH Parameters.

**Joint**-*i*	$a_{i}\;[\text{mm}]$	$\alpha_{i}\;[\circ ]$	$\theta_{i}\;[\circ ]$	$d_{i}\;[\text{mm}]$
1	0	0	0	$d_{1}$ *
2	141	0	$\theta_{2}$ *	0
3	120	0	$\theta_{3}$ *	0
4	0	−90	$\theta_{4}$ *	−54.5
5	0	90	$\theta_{5}$ *	−40.4
6	45	0	−90	$d_{6}$ *

* (asterisk) mark shows the joint variable. $d_{i}$ for the prismatic joint and $\theta_{i}$ for the revolute joint.

#### 2.4.2 Inverse kinematics

To control the position of the end-effector directly, inverse kinematics calculation is needed. The form of reference coordinate frame in Eq. (1) is generalized to the Eq. (4) where $f^{x},f^{y},f^{z},o$ are the three unit vectors in x, y, z direction of world coordinate and the one origin point of the coordinate frame. The joint variables can be calculated by following Eqs. (6), (7), (8), (9), (10), (11), (12), (13), (14), (15), (16), (17), (18), (19), (20), (21).

(4) $$F=\left[\begin{array}{cccc} f^{x} & f^{y} & f^{z} & o\\ 0 & 0 & 0 & 1 \end{array}\right]\text{;}\;f^{x},f^{y},f^{z},o\in R^{3\times 1}$$

(5) $$f={\left[\begin{array}{ccc} x & y & z \end{array}\right]}^{T}$$

The targeted end-effector position is denoted as $F_{E}^{T}$. The inverse kinematics calculation starts by finding the last joint coordinate ($F_{6}$) to satisfy the $F_{E}^{T}$ constraint in Eq. (6). Then, the x unit vector (axis) of $F_{5}$ can be found by Eq. (7) so that the angle between the $f_{5}^{x}$ and $f_{6}^{x}$ can be calculated in Eq. (8). Since the $F_{2}$ is coincident with the world coordinate, then the $f_{2}^{z}$ is the z-unit vector of world coordinate, i.e. ${\left[\begin{array}{ccc} 0 & 0 & 1 \end{array}\right]}^{T}$. It should also be noted that the last prismatic joint ($d_{6}$) was designed for the flexibility in acquiring the focus point of the image. Therefore on this work, the $d_{6}$ and $d_{f}$ values are fixed and known prior the inverse kinematics calculation, e.g., $d_{6}=d_{f}=0$. The values can be considered as the image-acquisition setting parameter.

(6) $$F_{6}=F_{E}^{T}{\left(\left[\begin{array}{cccc} 1 & 0 & 0 & 0\\ 0 & 1 & 0 & -\left|a_{6}\right|\\ 0 & 0 & 1 & d_{6}\\ 0 & 0 & 0 & 1 \end{array}\right]\left[\begin{array}{cccc} 1 & 0 & 0 & 0\\ 0 & 1 & 0 & 0\\ 0 & 0 & 1 & d_{f}\\ 0 & 0 & 0 & 1 \end{array}\right]\right)}^{-1}$$

(7) $$f_{5}^{x}=f_{6}^{y}\times f_{2}^{z}$$

(8) $$\theta_{5}=\frac{f_{5}^{x}\cdot f_{6}^{x}}{\left\|f_{5}^{x}\right\|\cdot \left\|f_{6}^{x}\right\|}$$

The origin point of joint coordinates $F_{5}$ and $F_{4}$ is calculated by Eqs. (9), (10). In order to find the $\theta_{4}$, the $o_{4}$ should be projected to the $f_{5}^{z}$ by the projection function $P\left(\cdot \right)$ in Eq. (11) to form a triangle in the x-y plane of the world coordinate (see Fig. 4). Then, $\theta_{4}$ can be calculated by rotating the vector $o_{4}-o_{4}^{f_{2}^{z}}$ with ψ angle, following Eqs. (13), (14), (15). The projection function $P\left(\cdot \right)$ has three arguments i.e., f the projected point, $f^{\mathrm{dir}}$ the direction vector, $f^{0}$ the origin point of the line segment where the projection occurs.

(9) $$o_{5}=o_{6}+\left|d_{5}\right|f_{6}^{y}$$

(10) $$o_{4}=o_{5}+\left|d_{4}\right|f_{2}^{z}$$

(11) $$P\left(f^{0},f^{\mathrm{dir}},f\right)=f^{0}+\left(f\cdot f^{\mathrm{dir}}-f^{0}\cdot f^{\mathrm{dir}}\right)\frac{f^{\mathrm{dir}}}{\left\|f^{\mathrm{dir}}\right\|}$$

(12) $$o_{4}^{f_{2}^{z}}=P\left(\left[\begin{array}{ccc} 0 & 0 & 0 \end{array}\right],f_{2}^{z},o_{4}\right)$$

(13) $$\psi =\cos^{-1}\left(\frac{{\left(a_{3}\right)}^{2}+{\left\|o_{4}^{f_{2}^{z}}-o_{4}\right\|}^{2}-{\left(a_{2}\right)}^{2}}{2a_{3}\left\|o_{4}^{f_{2}^{z}}-o_{4}\right\|}\right)$$

(14) $$f_{4}^{x}=\left[\begin{array}{ccc} \cos\psi & -\cos\psi & 0\\ \sin\psi & \cos\psi & 0\\ 0 & 0 & 1 \end{array}\right]\frac{o_{4}^{f_{2}^{z}}-o_{4}}{\left\|o_{4}^{f_{2}^{z}}-o_{4}\right\|}$$

(15) $$\theta_{4}=\frac{f_{4}^{x}\cdot f_{5}^{x}}{\left\|f_{4}^{x}\right\|\cdot \left\|f_{5}^{x}\right\|}$$

The $\theta_{3}$ and $\theta_{2}$ joint variables can be calculated in similar way with $\theta_{5}$, that is by finding the x-unit vector of the related joint coordinates as shown in Eqs. (16), (17), (18), (19), (20). Since the $F_{2}$ is coincident with the world coordinate, the $f_{2}^{x}$ is ${\left[\begin{array}{ccc} 1 & 0 & 0 \end{array}\right]}^{T}$. Finally, the $d_{1}$ can be calculated as the distance from the projected $o_{4}$ point to origin point as shown in Eq. (21).

(16) $$o_{3}=o_{4}-\left|a_{3}\right|f_{4}^{x}$$

(17) $$o_{2}=P\left(\left[\begin{array}{ccc} 0 & 0 & 0 \end{array}\right],f_{2}^{z},o_{3}\right)$$

(18) $$f_{3}^{x}=\frac{o_{3}-o_{2}}{\left\|o_{3}-o_{2}\right\|}$$

(19) $$\theta_{3}=\frac{f_{3}^{x}\cdot f_{4}^{x}}{\left\|f_{3}^{x}\right\|\cdot \left\|f_{4}^{x}\right\|}$$

(20) $$\theta_{2}=\frac{\left[\begin{array}{ccc} 1 & 0 & 0 \end{array}\right]\cdot f_{3}^{x}}{\left\|f_{3}^{x}\right\|}$$

(21) $$d_{1}=\left\|o_{4}^{f_{2}^{z}}\right\|$$

### 2.5 Key aspects of the hardware

MicroEye can be used to support TWM activities in several settings:

• Tool wear monitoring with limited machining area.
• Tool wear monitoring with the miniaturized micro-milling machine.
• Tool wear monitoring with moving measurement device.
• Tool wear monitoring without tool detachment.
• Tool wear monitoring with budget limitation, e.g., for middle-low manufacturing sites, laboratories.

## 3 Design files

### 3.1 Design files summary

.

**Table 2:** Design files.

Design filename	File type	Open source license	Location of the file
Link1	3D Model (*.stl)	CC-BY-SA-4.0	https://doi.org/10.17632/w26x9yrbtm.2
Link2	3D Model (*.stl)	CC-BY-SA-4.0	https://doi.org/10.17632/w26x9yrbtm.2
Link3	3D Model (*.stl)	CC-BY-SA-4.0	https://doi.org/10.17632/w26x9yrbtm.2
Link4	3D Model (*.stl)	CC-BY-SA-4.0	https://doi.org/10.17632/w26x9yrbtm.2
Link5	3D Model (*.stl)	CC-BY-SA-4.0	https://doi.org/10.17632/w26x9yrbtm.2
Link6	3D Model (*.stl)	CC-BY-SA-4.0	https://doi.org/10.17632/w26x9yrbtm.2
CoverBottom	3D Model (*.stl)	CC-BY-SA-4.0	https://doi.org/10.17632/w26x9yrbtm.2
CoverFront	3D Model (*.stl)	CC-BY-SA-4.0	https://doi.org/10.17632/w26x9yrbtm.2
CoverTop	3D Model (*.stl)	CC-BY-SA-4.0	https://doi.org/10.17632/w26x9yrbtm.2
PillarFrontLeft	3D Model (*.stl)	CC-BY-SA-4.0	https://doi.org/10.17632/w26x9yrbtm.2
PillarFrontRight	3D Model (*.stl)	CC-BY-SA-4.0	https://doi.org/10.17632/w26x9yrbtm.2
PillarBackLeft	3D Model (*.stl)	CC-BY-SA-4.0	https://doi.org/10.17632/w26x9yrbtm.2
PillarBackRight	3D Model (*.stl)	CC-BY-SA-4.0	https://doi.org/10.17632/w26x9yrbtm.2
GapFiller	3D Model (*.stl)	CC-BY-SA-4.0	https://doi.org/10.17632/w26x9yrbtm.2
HoleGuard	3D Model (*.stl)	CC-BY-SA-4.0	https://doi.org/10.17632/w26x9yrbtm.2
Retainer	3D Model (*.stl)	CC-BY-SA-4.0	https://doi.org/10.17632/w26x9yrbtm.2
SpacerInside	3D Model (*.stl)	CC-BY-SA-4.0	https://doi.org/10.17632/w26x9yrbtm.2
SpacerOutside	3D Model (*.stl)	CC-BY-SA-4.0	https://doi.org/10.17632/w26x9yrbtm.2
ControllerBackPCB	PDF (*.pdf)	CC-BY-SA-4.0	https://doi.org/10.17632/w26x9yrbtm.2
ControllerFrontPCB	PDF (*.pdf)	CC-BY-SA-4.0	https://doi.org/10.17632/w26x9yrbtm.2
ControllerSchematic	PDF (*.pdf)	CC-BY-SA-4.0	https://doi.org/10.17632/w26x9yrbtm.2
FrontPanelSchematic	PDF (*.pdf)	CC-BY-SA-4.0	https://doi.org/10.17632/w26x9yrbtm.2
AcrylicTop	PDF (*.pdf)	CC-BY-SA-4.0	https://doi.org/10.17632/w26x9yrbtm.2
AcrylicLeft	PDF (*.pdf)	CC-BY-SA-4.0	https://doi.org/10.17632/w26x9yrbtm.2
AcrylicRight	PDF (*.pdf)	CC-BY-SA-4.0	https://doi.org/10.17632/w26x9yrbtm.2
AcrylicBack	PDF (*.pdf)	CC-BY-SA-4.0	https://doi.org/10.17632/w26x9yrbtm.2
FirmwareMicroEye	Arduino (*.ino)	GNU GPL	https://doi.org/10.17632/w26x9yrbtm.2
ModParser	C++ (*.h,*.cpp)	GNU GPL	https://doi.org/10.17632/w26x9yrbtm.2
WearDetector	Python (*.py)	GNU GPL	https://doi.org/10.17632/w26x9yrbtm.2
WearAnalyzer	Python (*.py)	GNU GPL	https://doi.org/10.17632/w26x9yrbtm.2
SingleDetection	Python (*.py)	GNU GPL	https://doi.org/10.17632/w26x9yrbtm.2
BatchDetection	Python (*.py)	GNU GPL	https://doi.org/10.17632/w26x9yrbtm.2

### 3.2 Design files description

.

• **Link1**: 3D-printed body for the first link that mounts the servo motor of the second joint and connects to the linear stage of the first joint.
• **Link2**: 3D-printed body for the second link that mounts the servo motor of the third joint and connects to the servo motor horn of the second joint.
• **Link3**: 3D-printed body for the third link that mounts the servo motor of fourth joint and connects to the servo motor horn of the third joint.
• **Link4**: 3D-printed body for the fourth link that mounts the servo motor of the fifth joint and connects to the servo motor horn of the fourth joint.
• **Link5**: 3D-printed body for the fifth link that mounts the stepper motor of sixth joint and connects to the servo motor horn of the fifth joint.
• **Link6**: 3D-printed body for the sixth link that mounts the microscope camera and connects to the stepper motor of the sixth joint through linear stage mounting.
• **SpacerInner**: 3D-printed body for the inner spacer between arm link and servo motor.
• **SpacerOuter**: 3D-printed body for the outer spacer between arm link and servo motor.
• **ControllerBackPCB**: Template for printing the backside of controller unit PCB.
• **ControllerFrontPCB**: Template for printing the front side of controller unit PCB.
• **ControllerSchematic**: Wiring schematic of the controller unit PCB.
• **FrontPanelSchematic**: Wiring schematic of the front panel.
• **AcrylicTop**: Drawing for top transparent acrylic.
• **AcrylicLeft**: Drawing for left transparent acrylic.
• **AcrylicRight**: Drawing for right transparent acrylic.
• **AcrylicBack**: Drawing for top transparent acrylic.
• **FirmwareMicroEye**: Arduino code to control the operation of MicroEye.
• **ModParser**: Modified C++ code for parsing joystick message by using USB Host Shield 2.0 [11].
• **WearDetector**: Python script to detect the tool wear.
• **WearAnalyzer**: Python script to analyze the tool wear.
• **SingleDetection**: Python script to detect the tool wear of single image.
• **BatchDetection**: Python script to detect the tool wear of images in a batch.

## 4 Build instructions

The building process of MicroEye consists of five major steps: (1) printing the 3D-printed components, (2) assembling the positioning modules, (3) printing the controller PCB, (4) wiring the actuators of the positioning modules to the controller unit, and (5) building the enclosure with a front panel interface. The building process needs hand tools such as screwdrivers and pliers to assembly the positioning modules.

### 4.1 Printing 3D-printed components

The CAD models of the whole system, including its positioning system, have been shown in Fig. 2. Table 2 at sub-Section 3.1 summarizes the download location for all the 3D-print files. Table 3 shows the prices and sources of the MicroEye components. In order to achieve the minimum 3D-printing quality for the system, the printing process should follow below settings:

• The filament material is PLA (Poly Lactic Acid).
• High infill, minimum 70%.
• A raft layer is necessary to keep the dimension accurate, especially for the large flat surface.

It should be noted that the different models and types of 3D printers may produce different accuracy in the dimension of the 3D-printed object. Adjusting the targeted dimension in the CAD model relative to the reference one prior to the printing process is needed to compensate for the inaccuracy of printing. The adjustment process should be based on the experiment with the 3D printer itself. Furthermore, the surface roughness of the 3D-printed object is often deteriorated by some excessive or improper melted filament. Therefore, post-printing treatment is necessary to ensure that the assembly process of mating features of the component (e.g., hole, pin, or two mating surfaces) can be accomplished. Mini-grinder or cutter knife can be used as a tool to remove the excessive or improper melted filament. However, a mini-grinder should be used with notice, i.e., the heat dissipation from the continuous contact between the 3D-printed surface and the grinding tool can melt the surface itself. Therefore, lift-up of the grinding tool should be performed regularly to maintain the contact temperature adequate.

**Table 3:** Bill of material.

**Designator**	**Component**	**Number**	**Cost per unit currency**	**Total cost**	**Source of materials**	**Material type**
Base	Base linear stage with stepper motor NEMA 17	1	$39.83	$39.83	https://www.tokopedia.com/depoinovasi-mala/linear-actuator-z-axis-cnc-work-area-30mm	Metal, PLA
AX12A	Servo motor Dynamixel AX12A	3	$44.90	$134.7	https://www.robotis.us/dynamixel-ax-12a	Other
XL320	Servo motor Dynamixel XL320	1	$21.90	$21.90	https://www.robotis.us/dynamixel-xl-320	Other
MSTEP	Stepper Motor Screw with Linear Nut	1	$10.89	$10.89	https://www.aliexpress.com/item/32887764314.html	Other
A4988	Stepper Motor Driver A4988	2	$0.95	$1.90	https://www.aliexpress.com/item/10000278156894.html	Other
Mega2560	Arduino Mega 2560	1	$8.99	$8.99	https://www.aliexpress.com/item/32864836449.html	Other
IC	IC 74LS241N	1	$0.39	$0.39	https://www.aliexpress.com/item/32912039141.html	Semi-conductor
Converter	Buck converter LM2596	1	$1.80	$1.80	https://www.aliexpress.com/item/1005001507958453.html	Other
PSU	Power supply unit 12 V 120 W	1	$11.33	$11.33	https://www.aliexpress.com/item/1005002843829663.html	Other
PLA	3D Printer Filament PolyLactid 1 kg	1	$26.20	$26.20	https://www.aliexpress.com/item/33036735576.html	PolyLactid
Acrylic	Transparent Acrylic 3 mm × 300 mm × 300 mm	1	$11	$11	https://www.aliexpress.com/item/32833660352.html	Acrylic
Glue	Cyanoacrylate Glue	1	$1.64	$1.64	https://www.aliexpress.com/item/1005002570287090.html	Cyano-acrylate
XL320 Cable	Dynamixel Cable 3P-XL 130 mm	1	$2.78	$2.78	https://www.robotis.us/robot-cable-3p-xl-130mm-5pcs/	Other
AX12A Cable	Dynamixel Cable-3P 200 mm	3	$2.78	$4.32	https://www.robotis.us/robot-cable-3p-200mm-10pcs/	Other
B5R10	Bolt M5 Rounding Slotted Head 10 mm	4	$0.5	$2	https://www.aliexpress.com/item/32986027212.html	Metal
B3R8	Bolt M3 Rounding Slotted Head 8 mm	10	$0.5	$5	https://www.aliexpress.com/item/32986027212.html	Metal
B3F8	Bolt M3 Flat Slotted Head 8 mm	4	$0.5	$2	https://www.aliexpress.com/item/32983248803.html	Metal
B3H10	Bolt M3 Hex Head 10 mm	30	$0.166	$4.98	https://www.aliexpress.com/item/32968483467.html	Metal
B2R10	Bolt M2 Rounding Head 10 mm	4	$0.5	$2	https://www.aliexpress.com/item/32986027212.html	Metal
B2R5	Bolt M2 Rounding Head 5 mm	20	$0.5	$10	https://www.aliexpress.com/item/32986027212.html	Metal
N3H	Nut M3 Hexagon	4	$0.5	$2	https://www.aliexpress.com/item/32977174437.html	Metal
N2H	Nut M2 Hexagon	12	$0.5	$6	https://www.aliexpress.com/item/32977174437.html	Metal
LED8MM	LED 8 mm	3	$0.056	$0.168	https://www.aliexpress.com/item/4000312649149.html	Other
LED5MM	LED 5 mm	1	$0.014	$0.014	https://www.aliexpress.com/item/1005002603995042.html	Other
BUTTONP	Push Button	1	$0.256	$0.256	https://www.aliexpress.com/item/4000033026357.html	Other
BUTTONM	Momentary Button	1	$0.256	$0.256	https://www.aliexpress.com/item/32790281588.html	Other
USBHOST	USB Host Shield	1	$7.22	$7.22	https://www.aliexpress.com/item/1752058496.html	Other
Joystick	Joystick	1	$42.12	$42.12	https://www.aliexpress.com/item/1005002302690715.html	Other
Camera	Dinolite Microscope Camera AD4113ZT	1	$510	$510	https://www.alibaba.com/product-detail/TaiWan-Dino-lite-AM4113ZT-usb-portable_1600173784345.html	Other

### 4.2 Building the base

**Fig. 5:**
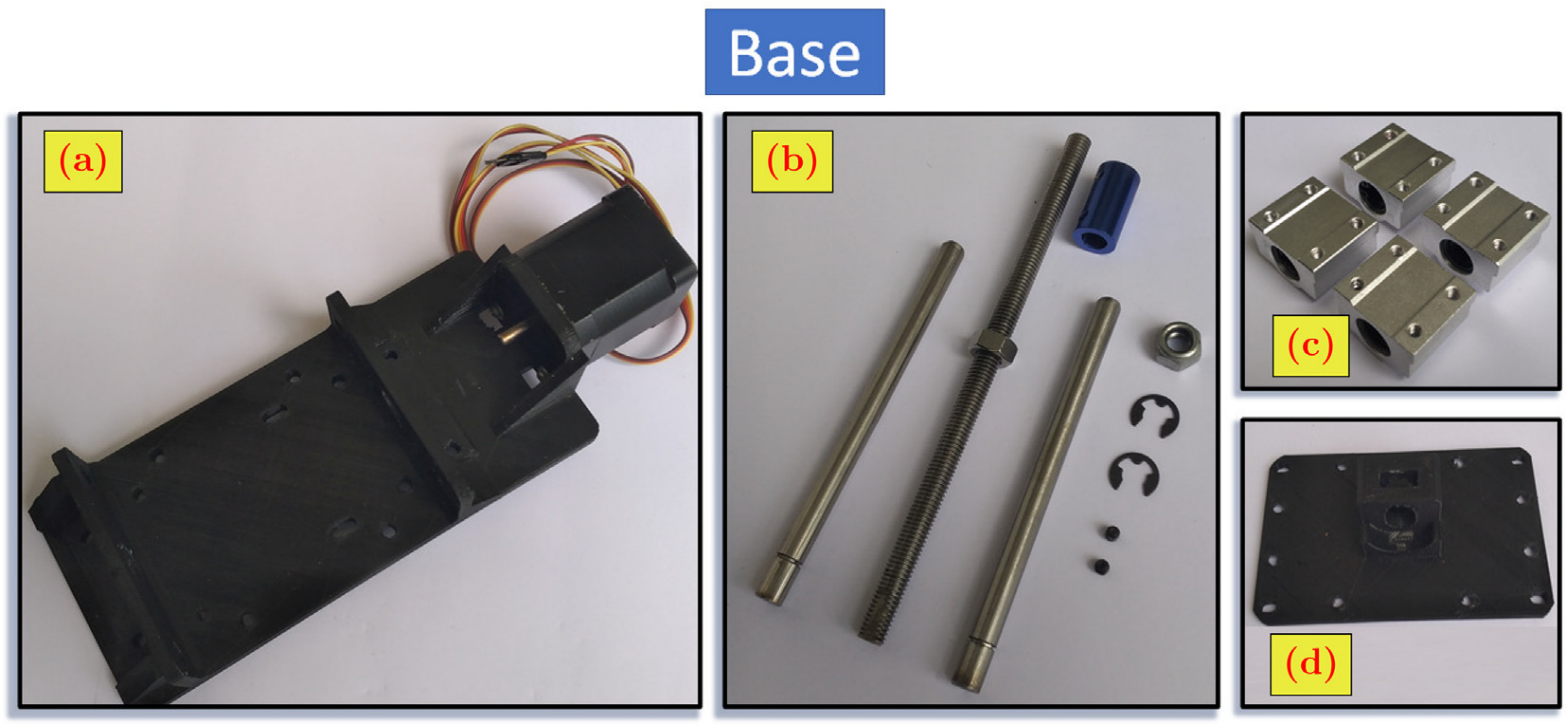
The components of base.

In this work, the base of the positioning system was a commercial product for Computerized Numerical Control (CNC) linear actuator, which is available at the online marketplace. However, most of the base parts are common commercial products available in the market, excluding the 3D-printed bodies. Fig. 5.a shows the stepper motor attached to the base 3D-printed body. Fig. 5.b shows the guiding rods, lead screw, lead screw nut, coupling, coupling nut, lock nut, and retaining rings. Fig. 5.c shows the linear ball bearings. Fig. 5.d shows the linear stage. The following steps are the assembly process of the base positioning system.

1. Insert the lead screw nut to the slot space below the linear stage (Fig. 6.a).

**Fig. 6:**
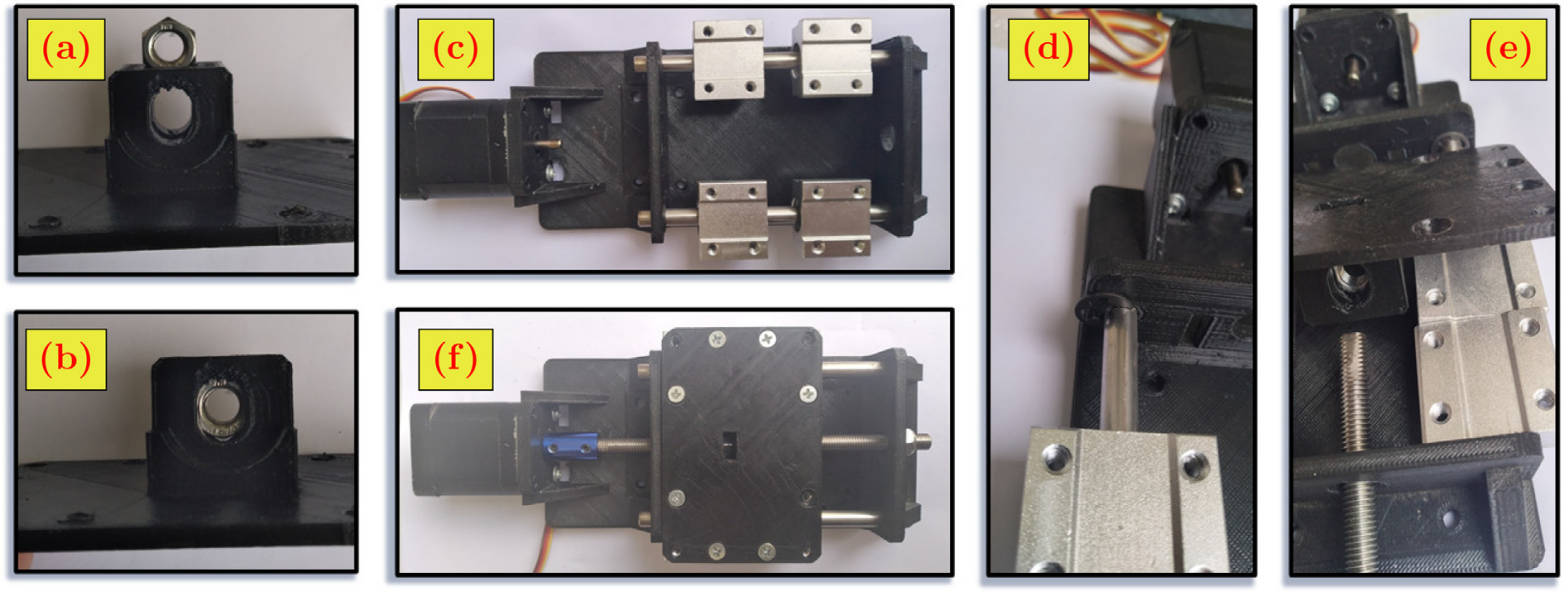
Assembly steps of base.
2. Hit the outer part of the lead screw nut with a hammer so that the nut is forced and located in the middle of the slot hole (Fig. 6.b).
3. Insert the guiding rods into the linear ball bearings. Each rod has two ball bearings on it.
4. Insert the guiding rods altogether with the linear bearings into the supporting holes in both sides of the base body (Fig. 6.c). From the four holes available, two holes are through-hole (THRU HOLE) for the entrance of the guiding rods.
5. Put the retaining rings to the small gap near the one end of the rods. The retaining ring prevents the rod’s motion in the axial direction. (Fig. 6.d).
6. From the right side (of the Fig. 6.f), insert the lead screw through the middle hole of base body and mate it with the lead screw nut in the linear stage as seen in Fig. 6.e.
7. Turn the lead screw until its most front end is about to hit the end of the stepper motor shaft.
8. Connect the lead screw and stepper motor shaft with coupling and fasten the connection with the coupling nut.
9. Insert the lock nut to another end of the lead screw and turn it towards the edge of the guiding rods support (Fig. 6.f).
10. Screw the linear stage to the four linear ball bearings (Fig. 6.f).

### 4.3 Building first link

**Fig. 7:**
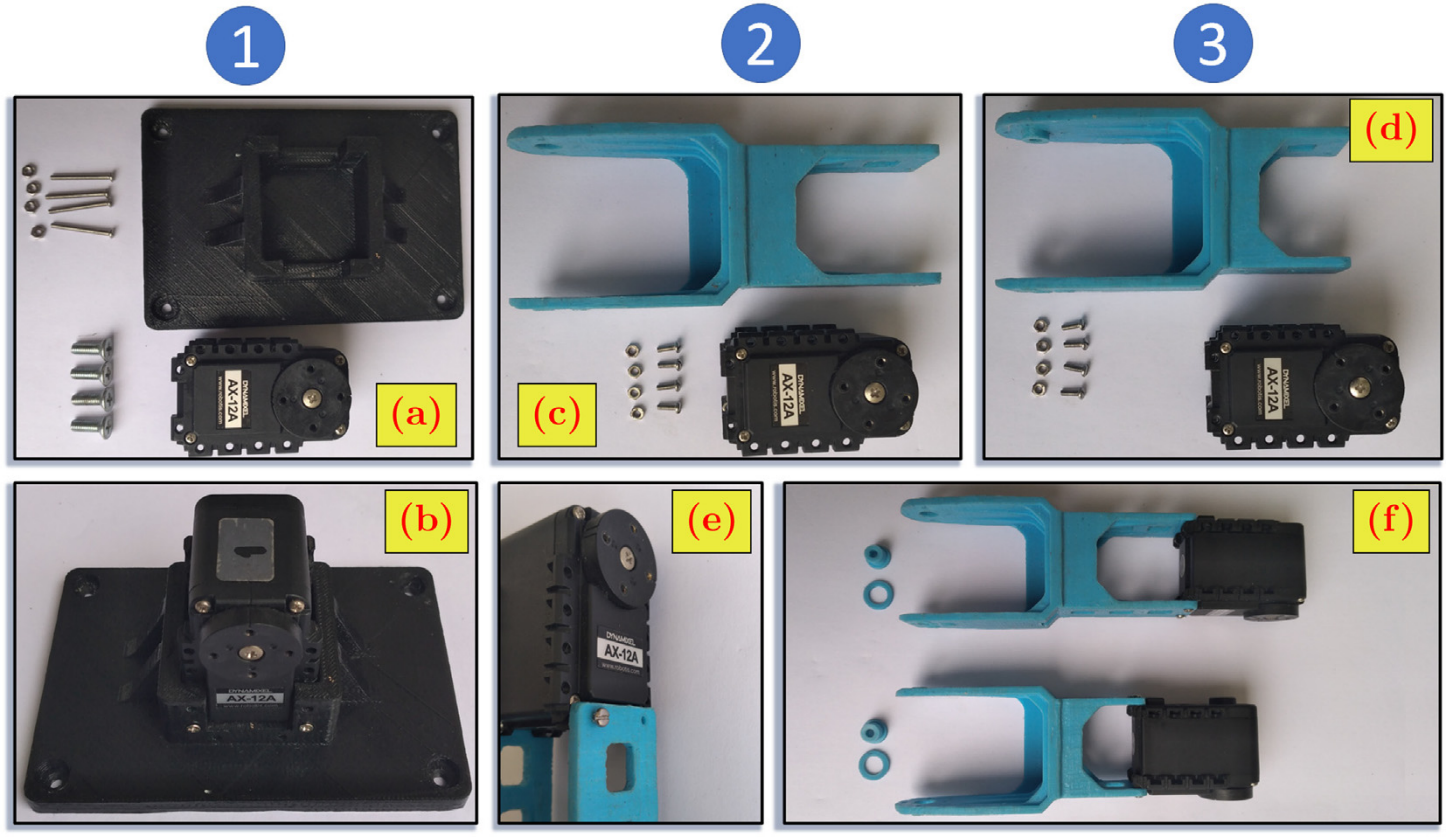
Assembly steps of first, second, and third links.

Fig. 7.a shows the first link 3D-printed body, first link servo motor (AX12A), M5 rounding slotted head 10 mm (B5R10) bolts, M2 rounding head 10 mm (B2R10) bolts, and M2 hexagon (N2H) nuts. The following steps are the assembly process of the first link.

1. Place the AX12A to the half pocket in the middle of the first link body (Fig. 7.b).
2. Fix the AX12A position by joining the B2R10 bolts with N2H nuts through holes at the wall of the servo motor pocket.

### 4.4 Building the second and third links

The 3D-printed body for the second and third links is identical but differs in size and dimension. Therefore, the assembly steps for both links are the same. The Fig. 7.c and Fig. 7.d show M2 rounding head 5 mm (B2R5) bolts, M2 hexagon (N2H) nuts, the links 3D-printed bodies, and the servo motor (AX12A). The following steps of assembly are for both links and repeated consecutively.

1. Put the AX12A to the one end of the link 3D-printed body, the one with smaller gap that fits to the width of the AX12A.
2. Fix the AX12A position by joining the B2R5 bolts with N2H nuts through holes near the edge of the link body (Fig. 7.e).

The two spacers (big and small) at Fig. 7.f are for the later purpose when the whole system assembly is performed.

### 4.5 Building the fourth and fifth link

**Fig. 8:**
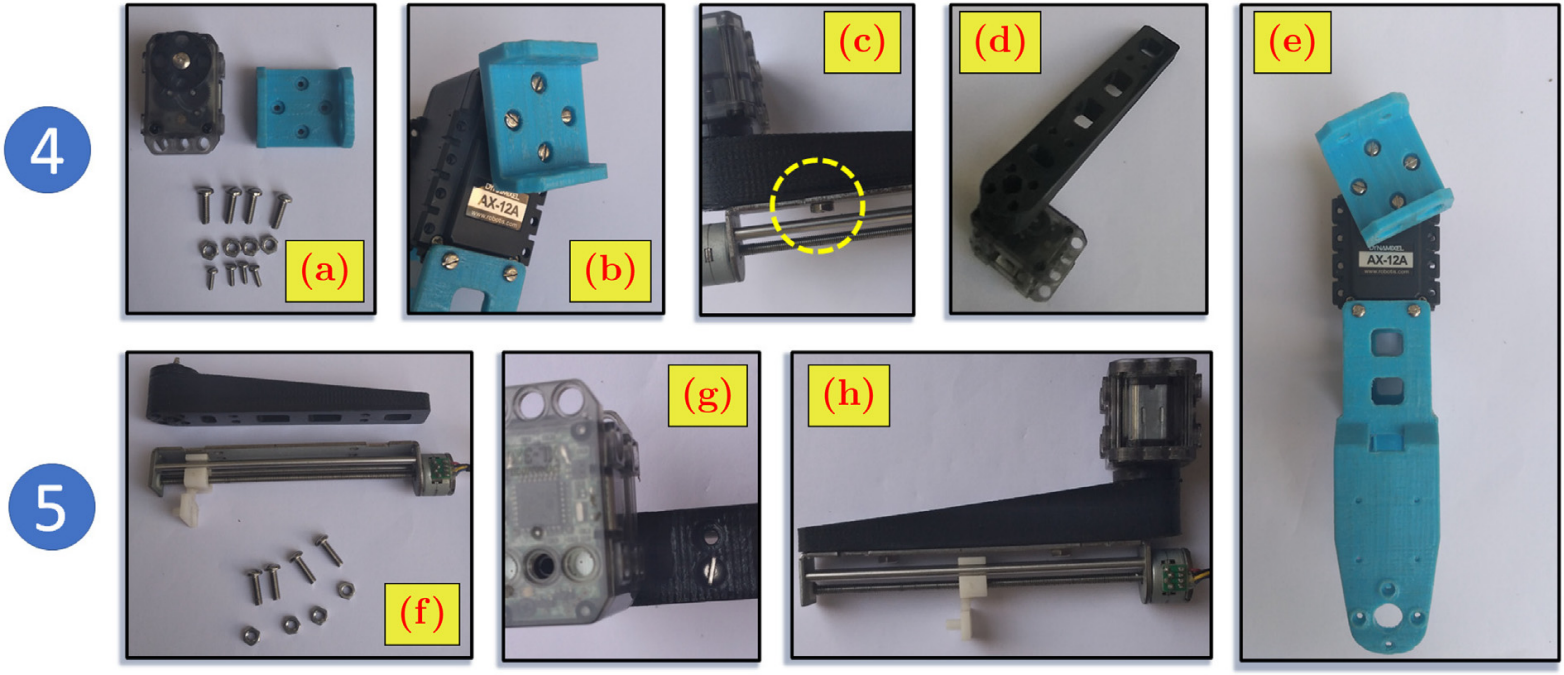
Assembly steps of fourth and fifth links.

Fig. 8.a shows the fourth link servo motor (XL320), fourth link 3D-printed body, M3 rounding slotted head 8 mm (B3R8) bolts, M2 rounding head 5 mm (B2R5) bolts, and M3 hexagon (N3H) nuts. Fig. 8.f shows micro-stepper motor (MSTEP), fifth link 3D-printed body, M3 rounding slotted head 8 mm (B3R8) bolts, and M3 hexagon (N3H) nuts. Differently from the assembly steps of previous links, the fourth and fifth links are built concurrently, with both parts used in a mixed manner.

1. Screw the fourth link 3D-printed body to the horn (mounting) of the servo motor (AX12A) from the result of the third link assembly (Fig. 7.f) by using B2R5 bolt. The result can be seen at Fig. 8.b and Fig. 8.e.
2. Screw the fifth link 3D-printed body to the horn of the XL320 by using B2R5 bolts (Fig. 8.d).
3. Attach the micro-stepper motor (MSTEP) to the fifth link 3D-printed body by using B3R8 bolt and N3H nut. Due to the very narrow space of the nut location, it is suggested to use a tweezer to keep the N3H nut in place (Fig. 8.c and Fig. 8.g). The result can be seen in Fig. 8.h.

### 4.6 Building the whole positioning system

**Fig. 9:**
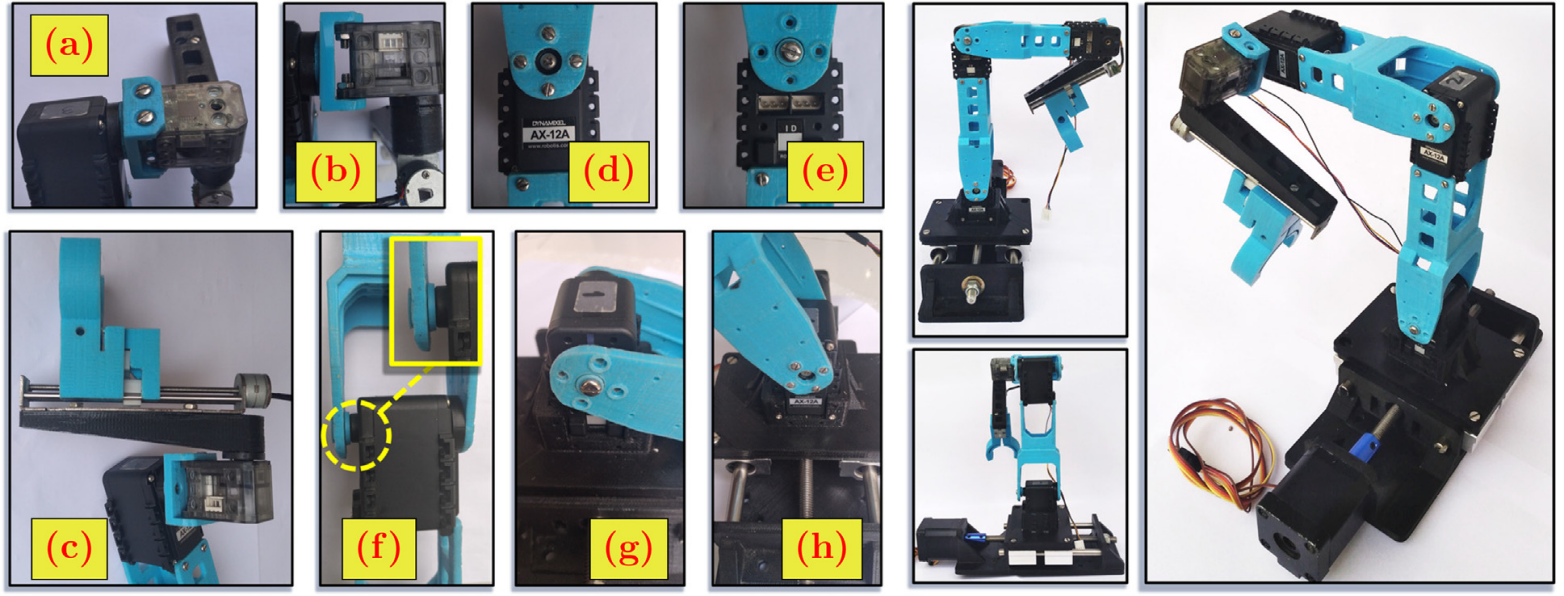
Building the positioning system module.

Five sub-modules of the positioning system have been built in the previous steps. The following steps explain the assembly process of the whole positioning system from its sub-modules (including the sixth link). The whole assembly of the positioning system module is shown in the most left figures of Fig. 9.

1. Mount the servo motor XL320 from Fig. 8.h to the 3D-printed body of fourth link in Fig. 8.e. by using B3R8 bolt and N3H nut (Fig. 9.a).
2. Use tweezer to keep N3H nut in narrow place (Fig. 9.b).
3. Attach (snap) the sixth link 3D-printed body to the mounting of the micro-stepper motor (MSTEP). The result can be seen in Fig. 9.c.
4. Screw the one end of the third link 3D-printed body from the Fig. 7.f to the mounting horn of AX12A motor in the second link by using B2R5 bolt, N2H nut, and B3R8 nut (Fig. 9.d and Fig. 9.e). To fill the gap between the motor and the link surface, the big and small spacers are located in pair, as seen in Fig. 9.f.
5. Similar to the previous step, screw the one end of the second link 3D-printed body from the Fig. 7.f to the mounting horn of AX12A motor in the first link, as seen in Fig. 9.g and Fig. 9.h.

### 4.7 Building the controller unit

Building the controller unit needs hand tools such as solder and cable cutter. Followings are the steps to build the controller unit:

1. Print the printed-circuit-board (PCB) according to the design file in Table 3.2 (Fig. 10.a).

**Fig. 10:**
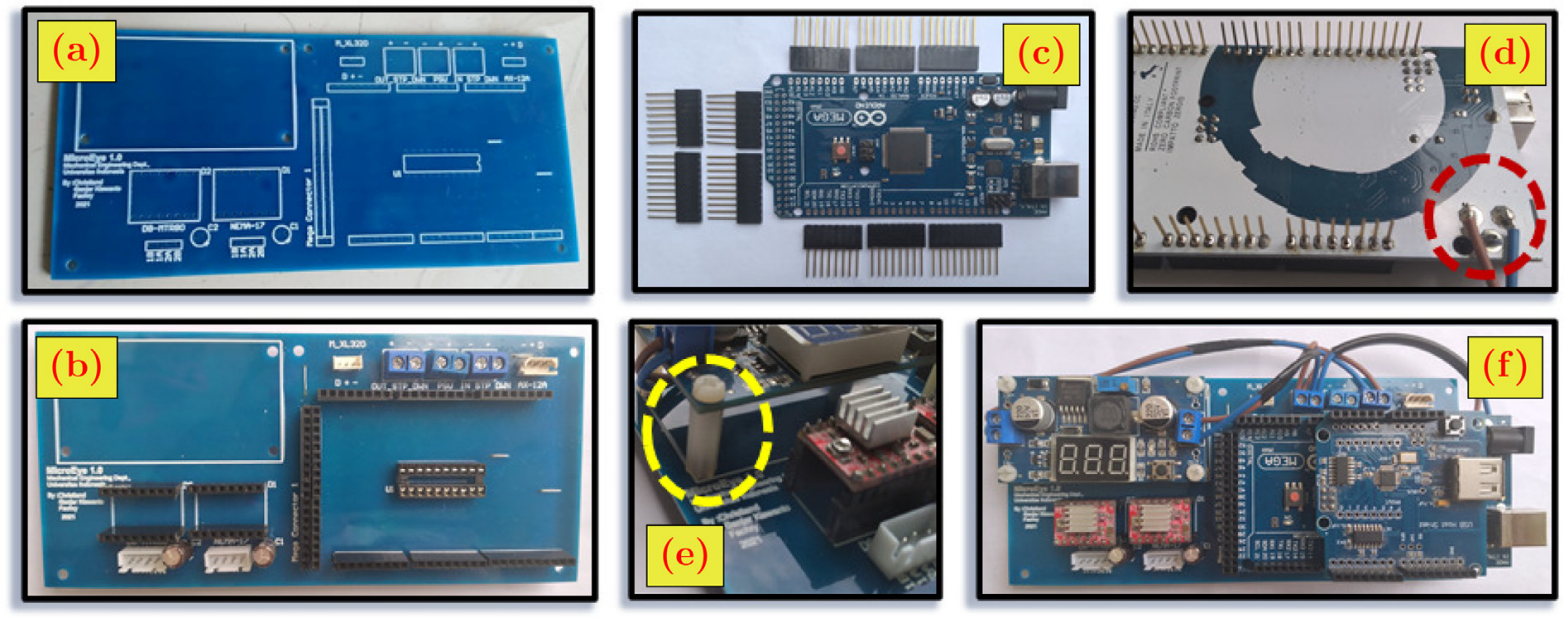
Building the controller unit.
2. Solder all the connectors, integrated circuit (IC) socket, capacitors, and headers to the PCB (Fig. 10.b).
3. Solder the stackable headers to the Arduino Mega 2560 (Fig. 10.c and Fig. 10.d)
4. Seat the motor driver modules, Arduino Mega 2560, USB shield module, and the buck converter to their designed headers (Fig. 10.e and Fig. 10.f). In order to level up the position of the buck converter, four M3 spacers are needed (Fig. 10.e).
5. Wire the input terminal of PCB (labeled as ”IN_STP_DOWN”) to the input terminal of the buck converter (labeled as ”IN”) by using a cable.
6. Wire the output terminal of PCB (labeled as ”OUT_STP_DOWN”) to the output terminal of buck converter (labeled as ”OUT”) and Arduino Mega 2560 jack altogether by using cables. Soldering the jack cable is needed on the Arduino Mega 2560 side (Fig. 10.d).
7. Wire the power terminal of PCB to output terminal 12 volt of the power supply using a cable.
8. Connect the power cable of the power supply to the electric source.
9. Tune the buck converter output to 8 volts by turning the adjuster pot.
10. Tune the current limit of motor driver A4988 by turning the adjuster pot so that the value of reference voltage (disregard the unit) is 0.5 times of the motor rated current; see more details in [12].
11. Disconnect the power cable of the power supply from the electric source.
12. Wire the three servo motors (1^st^, 2^nd^, 3^rd^ links) with the daisy-chain method by using Dynamixel cables. Later, the controller unit PCB is connected only to the first motor.
13. Plug each motor cable into the designed connector at PCB.

### 4.8 Building the controller enclosure and the front panel interface

**Fig. 11:**
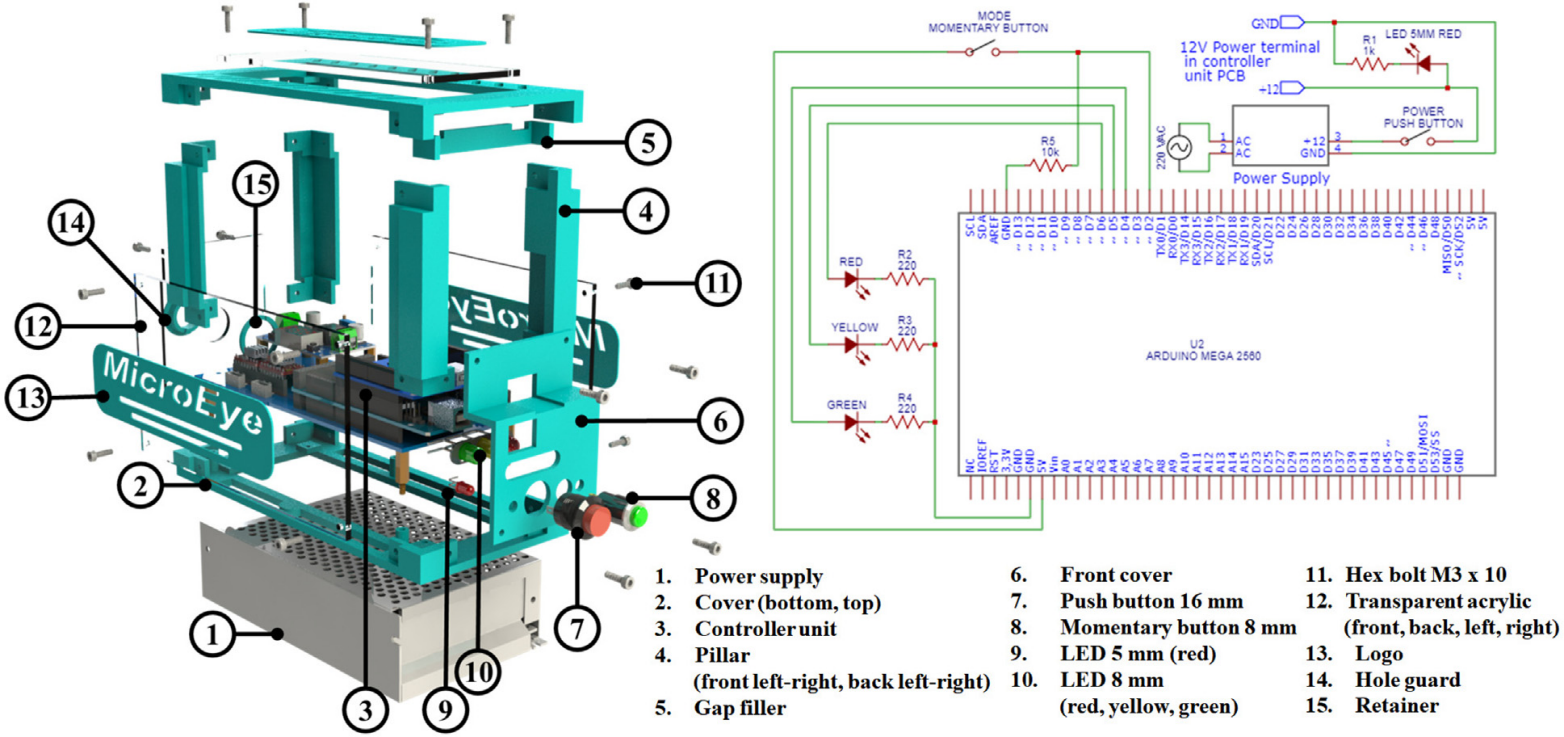
Enclosure assembly and front panel wiring schematic.

The enclosure protects the controller unit from unintended external disturbances and gives the user a convenient way to set up MicroEye. Fig. 11 shows the front panel wiring schematic and the exploded view of the enclosure assembly. Two major parts of the building steps are the wiring of the front panel interface and the assembly of the 3D-printed enclosure. The video of the assembly sequence is available in https://doi.org/10.17632/w26x9yrbtm.2 [13].

1. Wire the power button, mode button, power supply cable, actuators cables, and all the LEDs according to the schematic of the front panel (Table 2). Please ensure that all the wires are passed through the holes in the front cover, the hole guard, the retainer, and the hole at the back transparent acrylic (No. 6, 14, 15, and 12) before connecting to the Arduino Mega.
2. Affix the power supply (No. 1) to the bottom cover (No. 2) by using M3x10 bolts through the holes net of the power supply.
3. Seat the controller unit (No. 3) from the previous steps to the bottom cover (No. 2) using four M3x10 spacers.
4. Screw the four pillars (No. 4) altogether with the right and left transparent acrylics to the four corners of the bottom cover by using M3x10 bolts.
5. Snap the hole guard (No. 14) to its retainer (No. 15) through the middle hole in the back transparent acrylic (no. 12). Screw the back transparent acrylic to the back pillars by using M3x10 bolts.
6. Connect the necessary cables from the front panel and the actuators to the Arduino Mega and the PCB.
7. Attach all the buttons and LEDs to the front cover (No. 6), then screw the front cover to the front pillars using M3x10 bolts
8. Place and screw the gap filler (No. 5) to the top of the front cover and front pillars.
9. Screw the top cover to the top part of the pillars. Then, screw the top transparent acrylic to the top cover.
10. Glue the MicroEye logo (No. 13) to the center of the acrylic by using cyanoacrylate adhesive or strong double tape.

### 4.9 Software installation and setup

1. Install Arduino integrated development environment (IDE) [14].
2. Install Arduino libraries, i.e. Dynamixel Serial [15], Dynamixel XL-320 [16], USB Host Shield 2.0 [11].
3. Install DinoCapture microscope camera software [17].
4. Install Python programming [18].
5. Install OpenCV library for the Python programming environment [19].
6. Install Numpy library for the Python programming environment [20].
7. Install any Python integrated development environment (IDE), such as PyCharm [21].
8. Download ”FirmwareMicroEye.ino”, ”ModParser.cpp”, and ”ModParser.h” from the links provided in Table 2. Put those files under one folder named ”FirmwareMicroEye”.
9. Open the standard version of ”DynamixelSerial.h” and ”DynamixelSerial.cpp” files at the library folder of Dynamixel Serial. Find all the instances named *Serial* in those two files. Replace the *Serial* with *Serial2*. The edited version uses *Serial2* in Arduino Mega 2560 as the main connection between the library and the Dynamixel servo motor, so that the standard *Serial* communication can be freely used for monitoring purposes, i.e., by using *Serial.print()* function to show debugging messages.
10. Open ”FirmwareMicroEye.ino” in Arduino IDE and download (flash) it to the Arduino Mega 2560.

## 5 Operation instructions

**Fig. 12:**
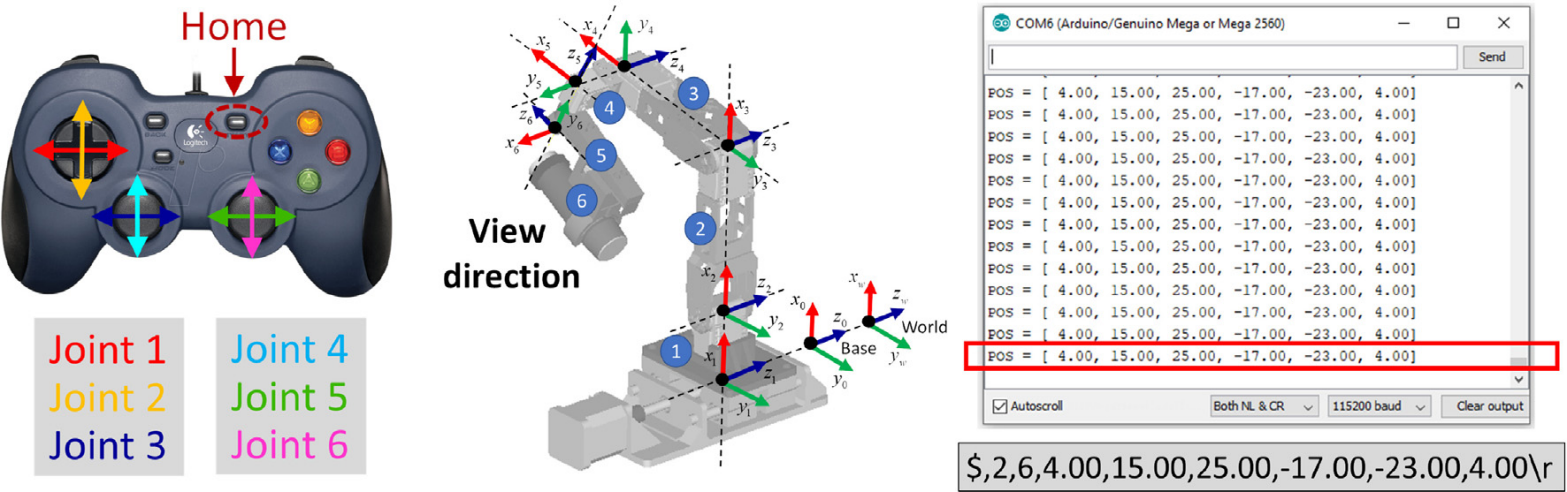
Control map of the joystick and the command message.

MicroEye can be operated by two means, i.e., direct control with joystick and serial communication from PC (video is available in https://doi.org/10.17632/w26x9yrbtm.2 [13]). Direct control with a joystick has the main role in the path planning of the end-effector. During the planning process, the path points of the end-effector are recorded in the form of sequential joint values (in degree unit for the revolute joint and millimeter unit for the prismatic joint). Later in the actual process of tool wear monitoring, the joint values from the record are executed sequentially to produce the motion. The joint values are sequentially executed for each joint (actuator) of MicroEye. On the other hand, operation with serial communication is provided to give the external parties (e.g., a PC that runs the process control of machining) access in controlling the MicroEye. The commands from the external parties must adhere to the protocol shown in Fig. 12. In that case, MicroEye runs as part of a bigger machining plan or sequence, e.g., when the tool wear monitoring by MicroEye is needed for every 20 min of the machining time in an actual micro-milling process. Followings are the standard procedure to operate the MicroEye:

### 5.1 Direct control with joystick

1. Place the MicroEye on the flat surface or using optional adjustable support (Fig. 13).

**Fig. 13:**
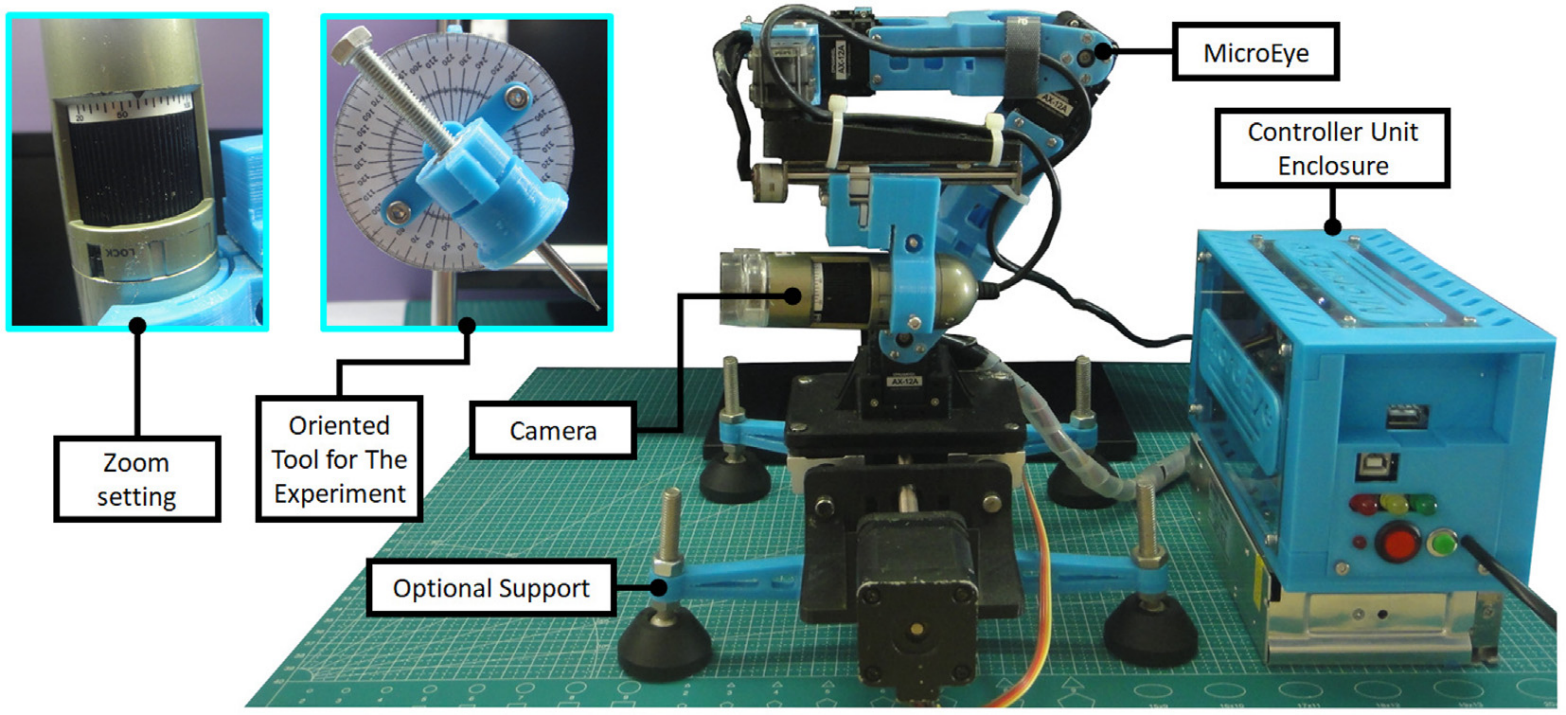
MicroEye and the oriented tool for the experiment.
2. Attach the microscope camera to its holder (the sixth link body).
3. Open DinoCapture software to capture the images from the microscope camera.
4. Plug the power cable of the controller unit to the electric source and turn on the MicroEye by pushing the red button until the red LED turns on (Fig. 13).
5. Plug the USB-type joystick into the USB port of the controller USB shield.
6. Ensure that the operation mode is manual joystick control indicated by the inactive yellow LED.
7. Move around the MicroEye by using the joystick to get the desired viewing angle of the camera. If the camera has shown the desired image quality, the values of joint variables can be recorded according to the protocol format. The values of joint variables are shown in the serial monitor window of Arduino IDE (Fig. 12). The control map of the joystick is shown in Fig. 12.
8. To perform the path planning, move the MicroEye to other positions and record all the values of joints variables sequentially.
9. When the camera is already positioned in the capture position, press the capture button at the DinoCapture software.

### 5.2 Operation through serial communication

1. Follow steps 1 to 4 from 6.1.
2. Push the mode button (green color Fig. 13) and make sure the green LED turns on. It means that the MicroEye is in serial communication mode.
3. Open the serial communication terminal software on the PC, e.g., built-in serial monitor tool in Arduino IDE or RealTerm [22].
4. Select the COM port that is associated to the USB cable of Arduino.
5. Establish the serial connection with 115200 bit-per second baud rate.
6. Upon succesful connection, send the formatted command message, e.g. ”$,2,6,4.00,15.00,25.00,-17.00,-23.00,4.00*\*r” to move the MicroEye.
7. When the camera is already positioned in the capture position, press the capture button at the DinoCapture software.

### 5.3 Tool wear detection

1. Open the “SingleDetection.py” or “BatchDetection.py” in the Python IDE. Those files are the example of the implementation of wear detection for single and multiple files.
2. Specify the filename (including the folder path) of the target and reference (wear template) images. It is assumed that the wear template has been made prior to the detection process.
3. Run the Python script (*.py). The result of detection will be shown after the detection process is completed.

## 6 Validation and characterization

### 6.1 Positioning

**Fig. 14:**
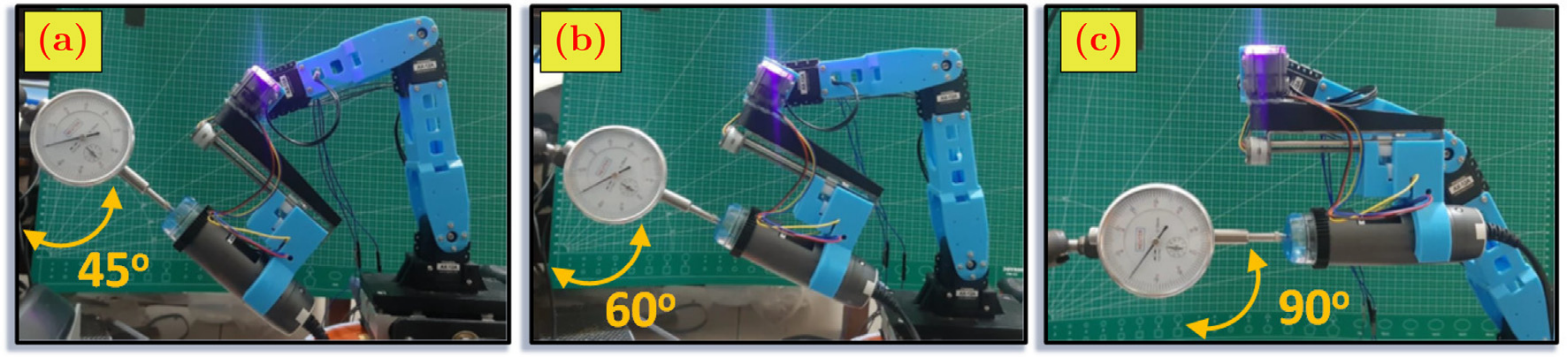
Linear position repeatability measurement by using dial gauge: (a) 45°, (b) 60°, (c) 90°.

The experiment has been conducted to validate and characterize the MicroEye performance based on: (A) motor angular position error, (B) motor angular position repeatability, (C) end-effector linear position error, and (D) end-effector linear position repeatability. The experiment was performed with two scenarios, i.e., with load and no-load at the end-effector. In every session, the MicroEye end-effector was moved to a particular position of the tool tip in the workspace by following reference configuration (Table 4). That position represented the position when MicroEye captured images of the tool surface. The tool axis is normal to the image plane of the camera. The tool axis orientation was set to the three values, i.e. 45°, 60°, and 90° with respect to vertical axis (similar to Fig. 14 without dial gauge). The camera attached to the end-effector is considered as the load for the MicroEye. The end-effector motion from the home position to the tool tip position was repeated ten times every session.

**Table 4:** Reference configuration.

**Tool**	$d_{1}^{r}$	$\theta_{2}^{r}$	$\theta_{3}^{r}$	$\theta_{4}^{r}$	$\theta_{5}^{r}$	$d_{6}^{r}$	$x_{\mathrm{end}}^{r}$	$y_{\mathrm{end}}^{r}$
45°	0	−6.397	−99.457	+60.853	0	0	4.640	−19.090
60°	0	−7.371	−85.037	+32.048	0	0	6.040	−18.020
90°	0	−27.343	−35.613	−27.044	0	0	9.410	−17.110

**Fig. 15:**
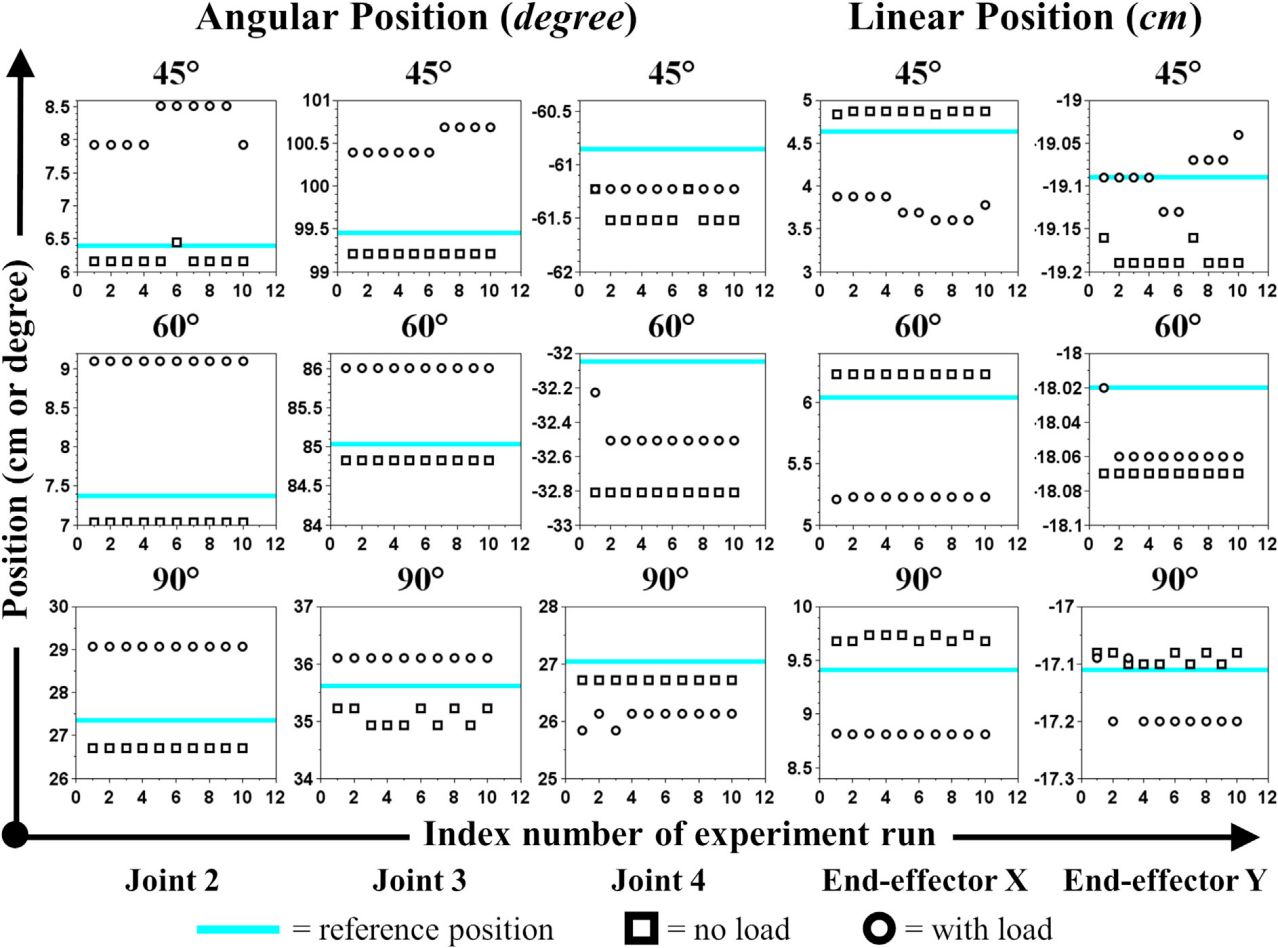
Angular and linear position at three tool orientations: 45°, 60°, 90°.

The series of measurement *p* at particular tool orientation *i* is formally defined as $P_{i}$ in Eq. (22), with n=10. The characterization parameters, i.e., error (ε) and repeatability ($\sigma^{2}$) of MicroEye were calculated for the 2^nd^ joint, 3^rd^ joint, 4^th^ joint, and end-effector from the *m* groups of measurements based on the tool orientation values. Joint angular position ($\theta_{2},\theta_{3},\theta_{4}$) and end-effector linear position ($x_{\mathrm{end}},y_{\mathrm{end}}$) were the variables of the system to be examined. After the end-effector reached the tool tip position, the error (ε) of angular position was measured by subtracting the last read angle (θ) with the reference angle ($\theta^{r}$) from each motor. The information of the last read angle from the motor’s built-in sensor is considered as the real angular position to calculate the real linear position (x,y) of the end-effector in the Cartesian coordinate by using the forward kinematics equation. Then, the linear position error was measured by subtracting the real linear position (x,y) with the reference linear position ($x^{r},y^{r}$). It should be noted that the angular position information from the built-in sensor in the motor has a measurement range from 0° to 300° with 1024 resolution (≈0.29°) [23]. The error and repeatability were calculated based on Eq. (23), (25). The repeatability is defined as the variance of the measurement range ($\overset{‾}{R}$) with the $d_{2}$ constant depending on the size (*n*) of measurements in one group. The $d_{2}$ constant is taken from appendix table VI in [24]. The data of angular and linear position from the experiment is shown in Fig. 15.

(22) $$P_{i}=p_{1},p_{2},\ldots ,p_{n}\;\text{;}\;\;\;\;n\in Z^{+},\;\;\;\;p\in [\theta_{2},\theta_{3},\theta_{4},x_{\mathrm{end}},y_{\mathrm{end}}]$$

(23) $$\varepsilon =\frac{1}{m\cdot n}\overset{m}{\underset{i=1}{\sum }}\overset{n}{\underset{j=1}{\sum }}\left(p_{j}-p^{r}\right)$$

(24) $$\overset{‾}{R}=\frac{1}{m}\overset{m}{\underset{i=1}{\sum }}\max(P_{i})-\min(P_{i})$$

(25) $$\sigma^{2}={\left(\frac{\overset{‾}{R}}{d_{2}\left(n\right)}\right)}^{2}$$

**Fig. 16:**
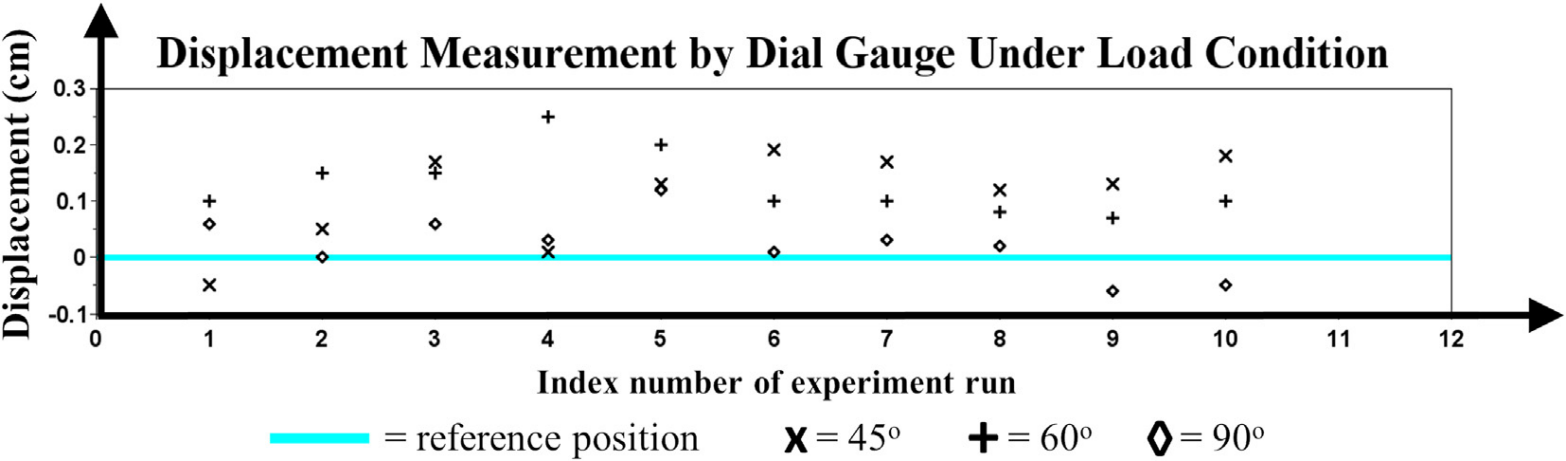
Displacement measurement by dial gauge at three tool orientations: 45°, 60°, 90°.

Besides using the latest information of angular position from the built-in motor sensor (experiment A, B, C), another calculation of error and repeatability under the load condition was performed using a dial gauge as the measurement tool. The linear displacement at some particular reference position (experiment D) based on three tool orientation values was repeatedly measured ten times. The dial gauge has a 0.01 mm resolution with a measurement range from 0 mm to 10 mm. Fig. 16 shows the measurement data for experiment D.

The summary of characterization parameters is shown in Table 5. Partial results of the experiment have also been presented in [25]. It can be seen from the result that the load at the end-effector contributes to the increase of the angular and linear positional error. That happened since the motors had already reached their maximum continuous torque when the load from the camera was applied. Furthermore, the more significant positional error happened for the lower-index joints (e.g., 2^nd^ joint compared to 3^rd^ joint). That happened since the motor of lower-index joints has to overcome the heavier load than the higher-index joints. Above all, the MicroEye achieved the angular position error as low as 0.596°, the linear position error as low as 0.0336 cm in the *x*-axis and 0.767 cm in the *y*-axis (≈0.767 cm in resultant). This maximum experimental accuracy is sufficient to ensure that the ROI (region of interest) image of the sub-millimeter-sized tool is viewable by the camera during the tool wear monitoring. Furthermore, the dial gauge measurement for positional accuracy under the load condition has confirmed that the position error was 0.0873 cm (one-tenth resultant of linear position error in experiment C).

**Table 5:** Experiment result.

**Exp.**	**Parameter**	**Unit**	**Symbol**	**No Load**	**Load**
A.	Motor angular position error (ε)	degree	$\varepsilon_{\theta_{2}}$ $\varepsilon_{\theta_{3}}$ $\varepsilon_{\theta_{4}}$	$-3.90\times 10^{-1}$ $-3.30\times 10^{-1}$ $-5.68\times 10^{-1}$	$1.75\times 10^{0}$ $8.38\times 10^{-1}$ $-5.96\times 10^{-1}$
B.	Motor angular position repeatability ($\sigma^{2}$)	degree	$\sigma_{\theta_{2}}^{2}$ $\sigma_{\theta_{3}}^{2}$ $\sigma_{\theta_{4}}^{2}$	$9.86\times 10^{-4}$ $3.14\times 10^{-2}$ $9.86\times 10^{-4}$	$4.04\times 10^{-3}$ $3.18\times 10^{-2}$ $3.90\times 10^{-3}$
C.	End-effector linear position error (ε)	cm	$\varepsilon_{x}$ $\varepsilon_{y}$	$2.36\times 10^{-1}$ $-4.13\times 10^{-2}$	$-7.67\times 10^{-1}$ $-3.36\times 10^{-2}$
D.	End-effector linear position repeatability ($\sigma^{2}$)	cm	$\sigma_{x}^{2}$ $\sigma_{y}^{2}$	$7.50\times 10^{-5}$ $2.90\times 10^{-5}$	$1.12\times 10^{-3}$ $6.76\times 10^{-4}$
E.	End-effector linear position error (ε) with dial gauge	cm	$\varepsilon_{\mathrm{dial}}$	–	$8.73\times 10^{-2}$
F.	End-effector linear position repeatability ($\sigma^{2}$) with dial gauge	cm	$\sigma_{\mathrm{dial}}^{2}$	–	$4.22\times 10^{-3}$

### 6.2 Image acquisition

**Fig. 17:**
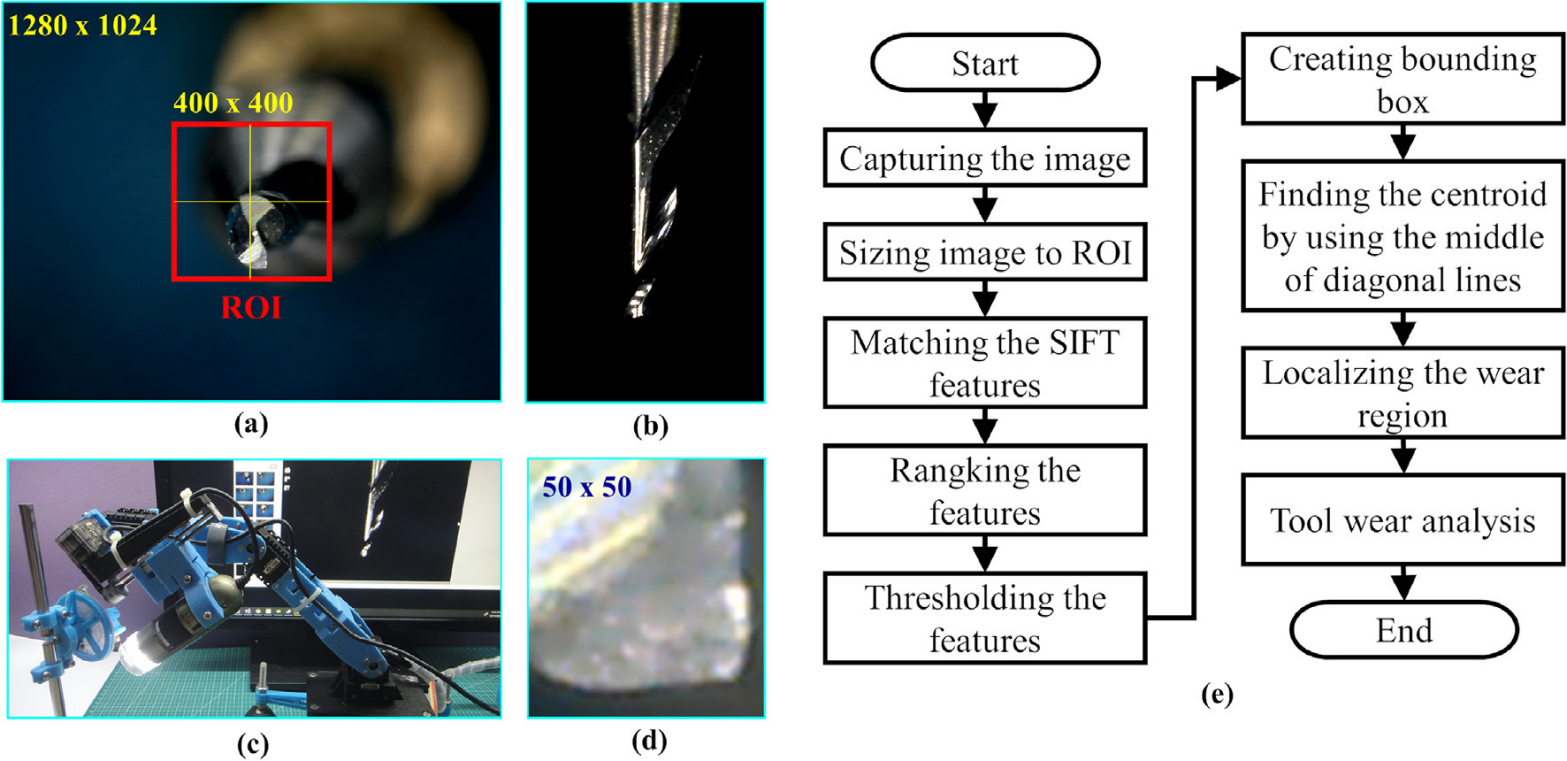
(a) Sample image of tool bottom from 45° tool orientation, (b) Sample image of tool flank side from 45° tool orientation, (c) Image acquisition position, (d) reference patch with 50 × 50 pixels size, and (e) flowchart of tool wear detection.

The experiment of image acquisition has been conducted with micro-tool wear as the object. The micro-tool has 1 mm cutting diameter, 3 mm shank diameter, 38 mm length, and two flutes. The micro-tool is for end-milling purposes. At the beginning of the experiment, the preparation step was conducted to set the distance between the end-effector (most outer part of the camera lens) and the micro-tool. The magnification dial on the camera was adjusted to get the image focus. The nominal distance and magnification setting were kept constant through all experiment runs. Sample image of the tool bottom is shown in Fig. 17.a. The original image size from the camera was set to 1280 × 1024 pixels. The region of interest (ROI) was set to 400 × 400 pixels located at the center of the original image. Within the ROI, the tool wear detection algorithm localizes the location of the wear region. The flowchart of the algorithm is shown in Fig. 17.d. In this wear detection algorithm, finding the features in the image I(x,y) were performed by using SIFT (Scale-Invariant Feature Transform) technique with OpenCV computer vision library [26], [27], [28]. The SIFT gave output a number of features as the tuple of keypoints (**k**) and descriptors (**d**), shown in Eq. 26. The BFM (Brute Force Matching) algorithm was used to find whether the reference features existed in the tested image by using the descriptors information [29]. The cost function showing the closeness (Δd) between the two descriptors was based on L1-norm distance (Eq. 27). The features were ranked and thresholded based on the closeness value. Only the top-20 best features at maximum were used to calculate the bounding box of the wear region. Then, the centroid of the wear region was calculated based on the middle point of the diagonal line of the bounding box.

(26) $$\left(k,d\right)=\mathrm{SIFT}\left(I(x,y)\right)$$

(27) $$\Delta d=\mathrm{BFM}\left(d,d^{r}\right)$$

**Fig. 18:**
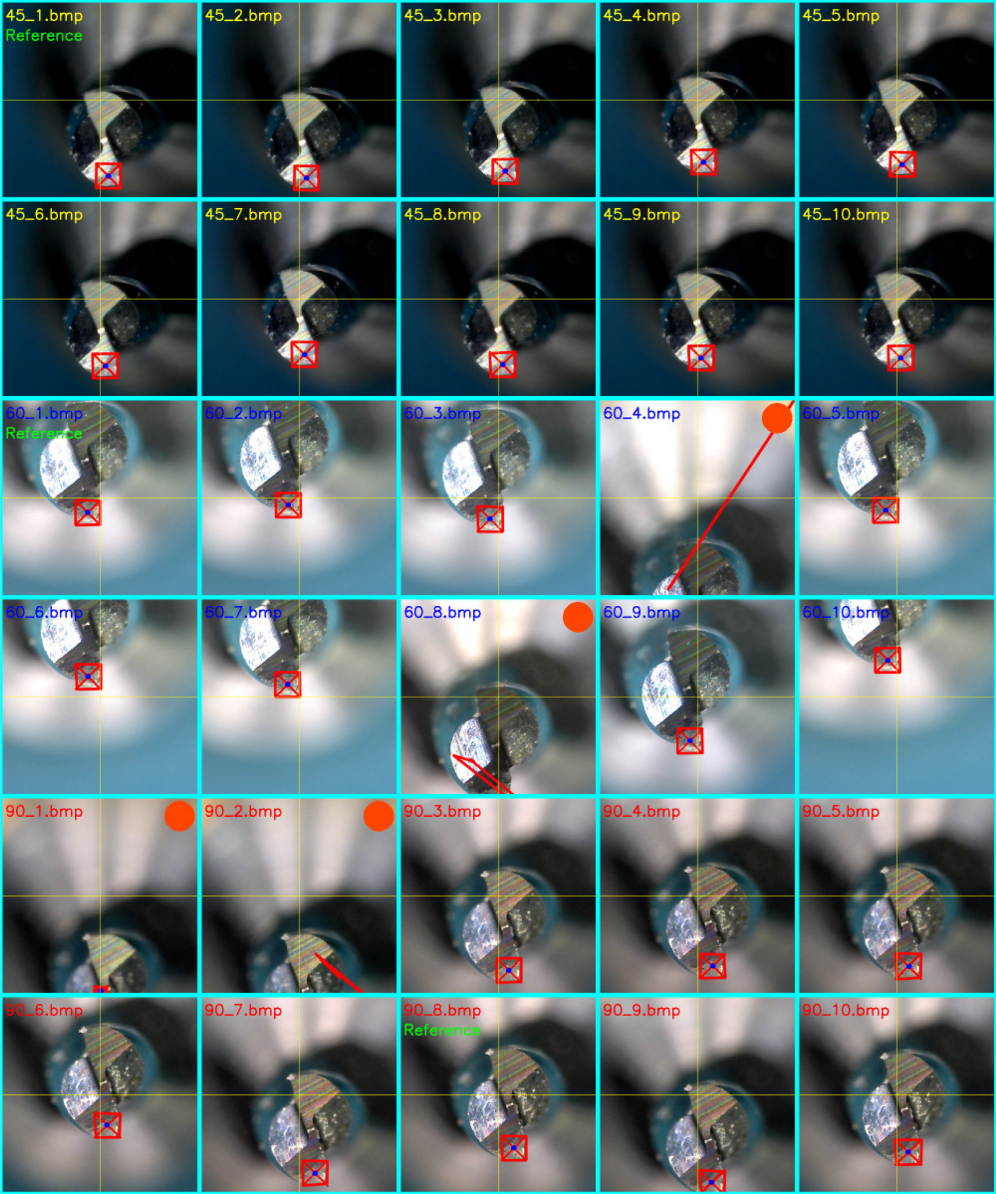
Image acquisition (ROI) from 45°, 60°, 90° tool orientations ( = bounding box of the detected wear region,  = centroid of the bounding box,  = failed detection due to the obstructed wear region.

The images of the tool bottom were captured from the three tool orientations as suggested in the Table 4 with ten repetitions (data set and video are available in https://doi.org/10.17632/w26x9yrbtm.2 [13]). From the series of tool images, the best image is selected manually as the reference image (labeled as ”reference” green text in Fig. 18). A 50 × 50 pixels patch containing the wear region was cropped from the reference image as the template for the reference features (Fig. 17.d). Later, the other images within one group of measurements were compared to the reference image. From the experiment result in Fig. 18, it is shown that MicroEye was capable of containing the full wear region to the inside of the ROI with 86.66% (26 out of 30) success rate. With the full wear region inside the ROI, MicroEye achieved 100% (26 out of 26) success rate in detecting the wear region. It is also shown in Fig. 18 that the position of the tool center was mostly shifted from the center of ROI. That issue was related to the inaccuracy of MicroEye positioning. Failed detection may also occur even though the wear region was fully inside the ROI. This issue can be related to the performance of the algorithm itself. The most probable cause was the considerably big change of light reflection (due to random positional error) that underperformed the features detection by SIFT. Above all, the MicroEye has achieved 86.66% success rate in detecting the tool wear region regardless of the actual positional accuracy.

### 6.3 Tool wear analysis

**Fig. 19:**
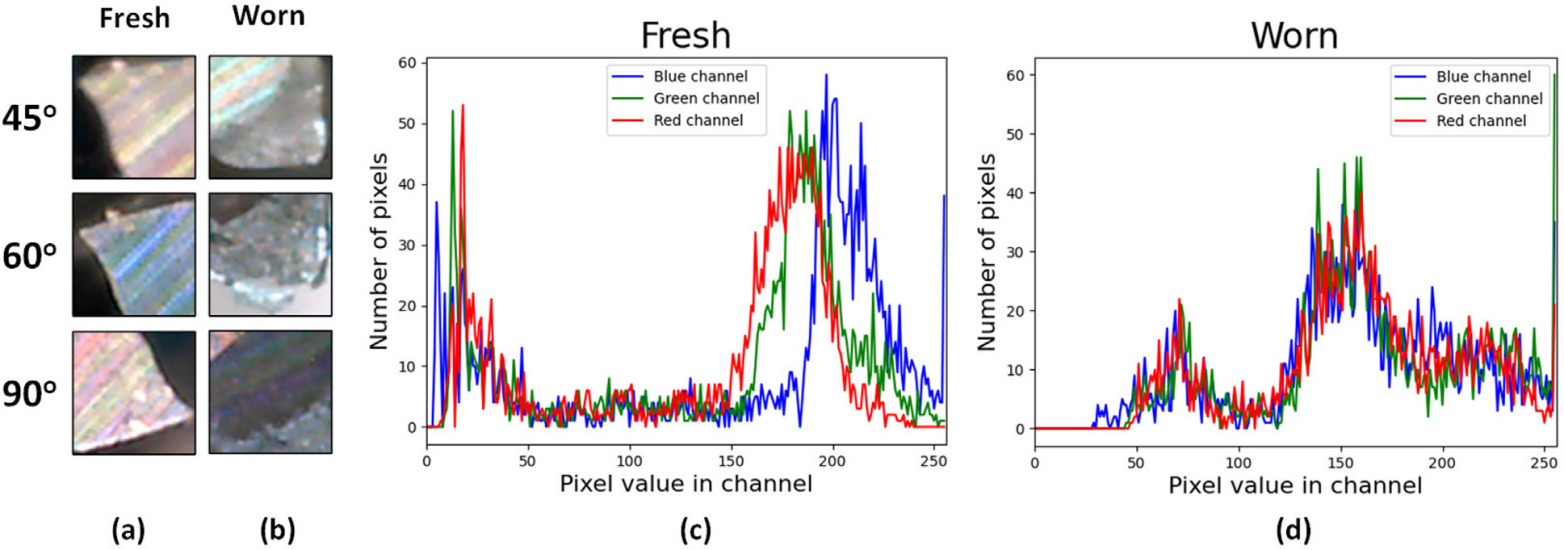
(a) The 50 × 50 pixels image of the fresh cutting edge at three tool orientations, (b) The 50 × 50 pixels image of the worn cutting edge at three tool orientations, (c) Histogram of three channels (RGB) of the fresh image at 45° tool orientation , (d) Histogram of three channels (RGB) of the worn image at 45° tool orientation.

The tool wear analysis was performed by comparing the detected wear region from the image acquisition step (sub-Section 7.2) and the fresh (newly unused) cutting edge image. The size of both images was set to 50x50 pixels. The wear region was automatically cropped from the original image by taking into account the centroid location of the detected wear region. The image’s histogram was the parameter to see the difference between the worn tool and the fresh tools. The quantitative wear measure can be inferred from the histogram difference of the wear region image with respect to the fresh cutting edge image. One reference image of the fresh cutting tool was dedicated for each tool orientation to accomodate the difference of light reflectance between the tool orientations. The reference image of the fresh cutting tool can be seen in Fig. 19.a.

**Fig. 20:**
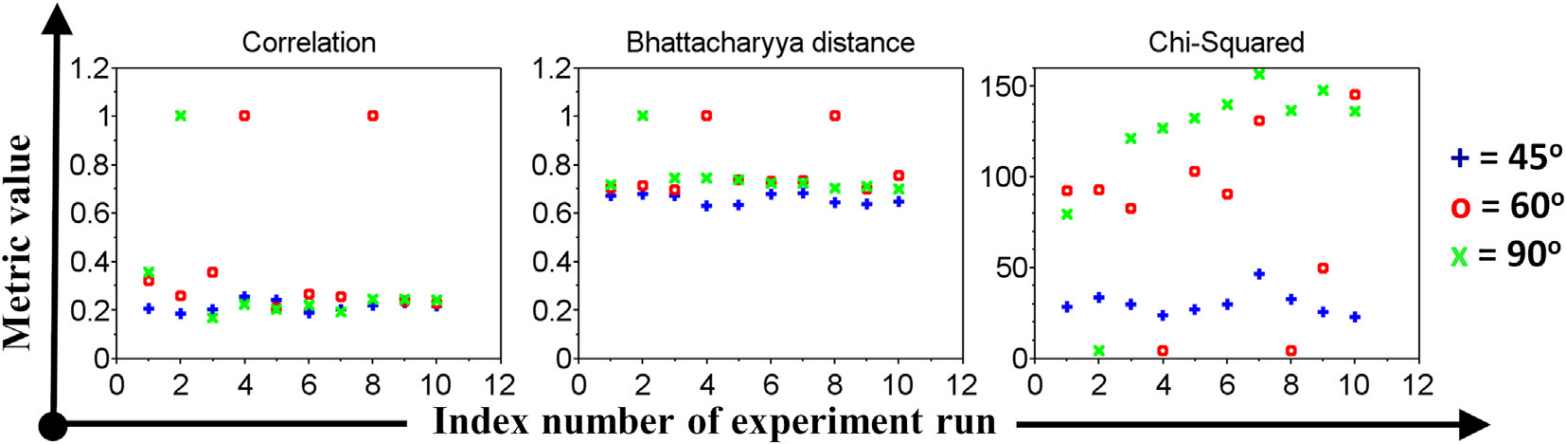
The histogram difference between the image of fresh cutting edge and the detected wear region.

In order to quantitatively measure the wear, three different metrics were used to calculate the histogram difference between images [30]. For the ”Correlation” metrics, a higher value (≈1) means that the two images are identical. Oppositely, for the ”Chi-squared” and ”Bhattacharyya distance” metrics, a lower value (≈0) shows the similarity between the two images. Fig. 20 shows that the most wear areas detected by the MicroEye can be consistently distinguished from the fresh cutting edge, e.g. ≈0.8 difference in ”Correlation” and ”Bhattacharyya distance” metrics value. Such metrics value can be considered as the changes of the cutting edge condition (due to the wear) relative to its initial condition. Thus, the wear quantification is possible for further analysis or decision. However, the histogram difference of the three undetected wear areas (such as 60_4.*bmp*, 60_8.*bmp*, and 90_2.*bmp* in Fig. 18) can not be measured correctly due to the off-frame (obstructed) wear region.

### 6.4 Concluding remarks

MicroEye has been developed as an alternative solution for tool wear monitoring in micro-milling applications. MicroEye offers a low-cost online TWM which uses 3D-printing technology and image-based wear detection and analysis. MicroEye experiment in positioning, image acquisition, and tool wear analysis shows that MicroEye can achieve reasonable accuracy and quality to capture the wear ROI area. In the aspect of the wear analysis, MicroEye was able to distinguish the degradation of the cutting edge condition due to the wear existence. In order to enhance the success rate of the wear detection, more advanced techniques (e.g., using deep learning technique) may be implemented to create an algorithm for wear detection and analysis that is robust to environmental influences (such as light reflection). Furthermore, the success rate of the wear detection may also be enhanced by improving the position accuracy of MicroEye. Minimally, the improved accuracy of the position can fully capture the wear region of the tool all the time. Improving the position accuracy may be achieved by using a more powerful motor to avoid reaching the saturated area of the continuous torque.

## Human and animal rights

None.

## Declaration of Competing Interest

The authors declare that they have no known competing financial interests or personal relationships that could have appeared to influence the work reported in this paper.
